# Circadian clock molecule REV-ERBα regulates lung fibrotic progression through collagen stabilization

**DOI:** 10.1038/s41467-023-36896-0

**Published:** 2023-03-09

**Authors:** Qixin Wang, Isaac Kirubakaran Sundar, Joseph H. Lucas, Jun-Gyu Park, Aitor Nogales, Luis Martinez-Sobrido, Irfan Rahman

**Affiliations:** 1 Department of Environmental Medicine, University of Rochester Medical Center, Rochester, NY USA; 2 Department of Internal Medicine, Division of Pulmonary, Critical Care and Sleep Medicine, University of Kansas Medical Center, Kansas City, KS USA; 3 Texas Biomedical Research Institute, Disease Intervention and Prevention Program, San Antonio, TX 78227 USA

**Keywords:** Respiratory distress syndrome, Translational research, Molecular biology

## Abstract

Molecular clock REV-ERBα is central to regulating lung injuries, and decreased REV-ERBα abundance mediates sensitivity to pro-fibrotic insults and exacerbates fibrotic progression. In this study, we determine the role of REV-ERBα in fibrogenesis induced by bleomycin and Influenza A virus (IAV). Bleomycin exposure decreases the abundance of REV-ERBα, and mice dosed with bleomycin at night display exacerbated lung fibrogenesis. Rev-erbα agonist (SR9009) treatment prevents bleomycin induced collagen overexpression in mice. Rev-erbα global heterozygous (Rev-erbα Het) mice infected with IAV showed augmented levels of collagens and lysyl oxidases compared with WT-infected mice. Furthermore, Rev-erbα agonist (GSK4112) prevents collagen and lysyl oxidase overexpression induced by TGFβ in human lung fibroblasts, whereas the Rev-erbα antagonist exacerbates it. Overall, these results indicate that loss of REV-ERBα exacerbates the fibrotic responses by promoting collagen and lysyl oxidase expression, whereas Rev-erbα agonist prevents it. This study provides the potential of Rev-erbα agonists in the treatment of pulmonary fibrosis.

## Abstract (web summary)

The molecular clock REV-ERBα regulates lung injury during fibrosis, but the role of REV-ERBα in fibrogenesis remains unknown. Here, the authors show that REV-ERBα interacts with the lysyl oxidase-collagen axis during fibrogenesis and demonstrate the therapeutic potential of Rev-erbα agonist against lung fibrosis.

## Introduction

Idiopathic pulmonary fibrosis (IPF) is a chronic interstitial lung disease characterized by progressive lung scar tissue formation that is typically accompanied by impaired lung function and difficulty breathing^1^. The onset of pulmonary fibrosis is usually initiated by the dysregulation of tissue repair mechanisms which can be induced by various causes, such as air pollution (asbestos), antineoplastic drugs, and respiratory viral infections such as influenza A virus (IAV) and even coronavirus (SARS-CoV-2) infection^2,3^. In previous decades, rigorous basic studies have improved our understanding of pro-fibrotic pathogenesis and developed many candidates for anti-fibrotic therapy. However, there are no effective therapeutics for IPF, and the detailed molecular mechanism of fibrogenesis is still poorly understood^4–6^.

Currently, nintedanib and pirfenidone are the only Food and Drug Administration (FDA)-approved drugs for the treatment of pulmonary fibrosis, which only serve to slow the progression of pulmonary fibrosis^7^. Investigating new promising molecular pathways involved in fibrogenic responses is urgently needed, and Rev-erbα has become a promising candidate^8,9^. REV-ERBα is a transcriptional repressor that regulates mRNA transcriptions involved in circadian rhythms, metabolism, and inflammatory responses^10–13^. Oscillations in circadian rhythm are controlled by the competition of two nuclear receptors, REV-ERBα, and retinoic acid-like orphan receptor alpha (RORα)^14^. REV-ERBα inhibits the transcription and translation of circadian locomotor output cycles kaput (CLOCK)/brain and muscle ARNT-like 1 (*BMAL1*, also known as *ARNTL*), which will form a heterodimer and bind to E-box and promote the transcription/translation of either core clock molecules or downstream targets^15^. For regulating BMAL1 and CLOCK expression, RORα competes with REV-ERBα to bind with ROR response elements (ROREs) to activate the transcription of BMAL1 and CLOCK^15^ forming an auto-feedback system with REV-ERBα and providing stability and precision to molecular clock regulation. Interestingly, the downstream gene targets of E-box include various fibrotic markers such as α-smooth muscle actin (αSMA) and vimentin (VIM)^16^. Moreover, the removal of REV-ERBα has been associated with increased risks of lung inflammation and premature senescence, which has been confirmed by our and others’ previous studies^17–19^.

Circadian clock molecules are identified as essential mediators of pulmonary injuries with various causes, such as cigarette smoke (CS) and IAV^20–23^. Previous studies have described the importance of circadian molecules in key cell subtypes, including club cells, alveolar macrophages, and fibroblasts, in the lung microenvironment in response to injury and inflammatory mediators^8,19,24,25^. Previous findings showed that CS exposure and IAV infection-induced lung injuries are associated with disruption of the circadian clock and impaired lung function, survival rate, and daily ambulatory activity^26,27^. Various studies to date demonstrate the fundamental interactions of core clock molecules, such as REV-ERBα or BMAL1, with lung inflammatory responses and the development of chronic obstructive pulmonary disease (COPD) by CS exposure^23^. Currently, only one study has shown that *REV*-*ERBα* deficiency in lung fibroblasts exaggerates bleomycin-induced lung fibrogenesis^8^. However, the mechanism and role of REV-ERBα in lung fibrogenesis via collagen synthesis and its regulation during IAV infection are not known. Stabilization of collagen fibers is regulated by lysyl oxidase, a copper-dependent amino oxidase, via crosslinking the extracellular matrix proteins (collagen and elastin), thereby preventing collagen degradation^28^.

Our previous study has identified the potential of REV-ERBα in regulating epithelial-mesenchymal transition (EMT) and fibroblast differentiation induced by CS and TGFβ^27^. We, therefore, hypothesize that REV-ERBα is important in regulating fibrotic progression in the lungs, by targeting collagen synthesis and its stabilization pathways.

Here we show, the abundance of REV-ERBα is decreased during fibrogenesis, and loss of REV-ERBα augments the fibrotic responses caused by IAV infection. Furthermore, enhanced REV-ERBα activity/abundance will reduce abnormal collagen accumulation by inhibiting the expression of lysyl oxidases during myofibroblast differentiation.

## Results

### Dysregulated protein abundance of REV-ERBα, COL1A1 and LOX were observed in IPF patients compared with healthy controls

**Fig. 1:**
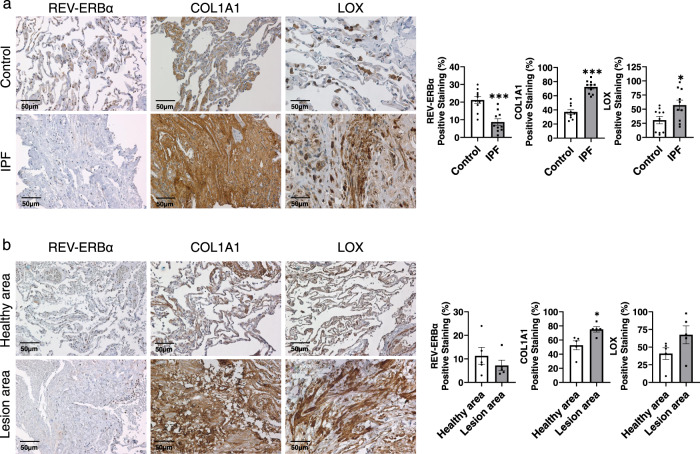
Decreased REV-ERBα protein abundance and increased protein levels of COL1A1 and LOX in IPF lungs compared to healthy control. Healthy control and IPF formalin fixed-paraffin embedded (FFPE) lung samples were purchased from Origene Inc. Healthy controls contained 100% normal lung architecture with 85% alveoli surface area. IPF patient samples contained at least 50% lesion surface area. The protein abundance of REV-ERBα, COL1A1, and LOX were visualized and determined by IHC. **a** The comparisons of protein distribution and abundance were performed between healthy control and IPF patient (*n* = 10 per group), **b** or between the healthy area and lesion area from the same IPF patient. The images were taken, and the positive stained area was calculated by ImageJ (*n* = 5 per group). Data were shown as mean ± SEM, unpaired *t*-test was used for **a** and **b**. Bar size: 50 µm. (*p < 0.05, ***p < 0.001; scale bar: 50 μm).

It is well known that excessive extracellular matrix (ECM) protein production occurs during fibrosis and is deposited within the lesion areas. Human lung sections with verified pathology were purchased from Origene Inc. All the healthy controls were within the normal limits with 100% normal area and at least 80% alveoli area, while the IPF samples were composed of at least 40% lesion area (Supplementary Table 1). As shown in Fig. 1a, we observed high expression of type 1 collagen (COL1A1) over the injured tissue area and elevated lysyl oxidase (LOX) protein in IPF patients compared with healthy controls. Both COL1A1 and LOX were highly expressed among ECM in the lesion tissues in IPF samples, whereas limited COL1A1 and Lox were expressed in healthy controls. Consistent with previous data, we observed the diminished protein abundance and distribution of REV-ERBα in the fibrotic lesions from IPF samples, whereas REV-ERBα was highly expressed in the nuclei of healthy controls with limited protein abundance observed in the cytoplasmic area. Similar results were observed in the healthy and lesion area in IPF samples as well (Fig. 1b). A decreased trend of REV-ERBα was found in the lesion area compared to the healthy sections, and upregulation of COL1A1 and LOX was found in the lesion area compared to the healthy area. Compared to the control groups, the protein abundance of REV-ERBα was decreased in the healthy area (Control: 21.294% vs. healthy area from IPF: 11.296%) from IPF samples, and slightly increased protein levels of COL1A1 (Control: 37.074% vs. healthy area from IPF: 52.604%) and LOX (Control: 30.439% vs. healthy area from IPF: 40.836%) in the healthy areas from IPF samples compared to control group were observed (Fig. 1). A previous study has identified that REV-ERBα is fundamental in IPF progression^8^, and we determined how Rev-erbα affects the development of pulmonary fibrosis.

### Circadian clock genes, including REV-ERBα were dysregulated in bleomycin-induced fibrosis

**Fig. 2:**
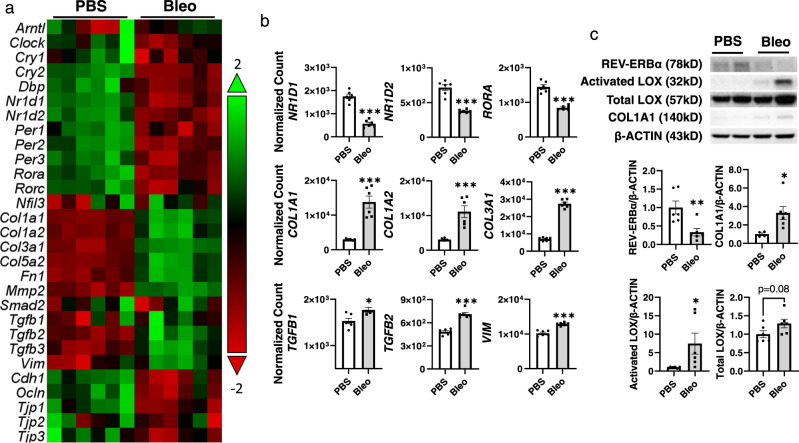
Altered circadian and profibrotic mRNA and protein expressions were observed in bleomycin induced fibrotic responses. Lungs from C57BL/6J WT mice (Combined male and female (*n* = 2–3 each) for analysis) dosed with bleomycin at day 14 were snap-frozen and used for RNA isolation. RNA isolated from lung homogenates were used to identify the circadian and profibrotic related gene expressions by our customized nanostring panel through nCounter SPRINT Profiler. The transcripts levels of RNA targets (Normalized Count) were normalized and visualized by nSolver software. **a** Dysregulated genes are shown as a heatmap with circadian genes on top and profibrotic genes on the bottom. **b** Selected gene expressions were shown as a bar graph (*n* = 6 mice per group). **c** Proteins isolated from lung homogenates were used to detect the abundance of REV-ERBα, LOX, Activated LOX, and COL1A1. Represented blots are shown here, and protein expression fold change was calculated based on the normalization of β-ACTIN (*n* = 4–6 mice per group). Data were shown as mean ± SEM, unpaired two-side *t*-test was used for **b** and **c**. (*p < 0.05, **p < 0.01, ***p < 0.001 vs. PBS).

To understand the expression of Rev-erbα and related circadian genes in the in vivo model of fibrosis, we treated C57BL/6J wild-type (WT) mice with bleomycin (1.5 units/kg) to induce fibrosis and determine the gene expression of circadian and fibrotic-related genes. According to previous studies, most of the fibrotic markers were dysregulated significantly at day 14, and there was no significant difference at day 14, 21, and 28 post-injury^29–31^. Another report also described that variable outcomes appeared after day 21 post-injury, and even recovered to baseline^32^. Hence, we have selected day 14 post-injury as our end-time point for bleomycin-induced lung injury. After 14 days of bleomycin-induced lung injury, we found decreased gene expression of *REV-ERBα* (Gene symbol: *NR1D1*), *REV-ERBβ* (Gene symbol: *NR1D2*), *RORα* (Gene symbol: *RORA*), *CLOCK*, *CRY1/2*, *PER1/2/3* and *DBP* (Fig. 2a, b and Supplementary Fig. 1). There was no change in gene expression of *BMAL1* (Gene symbol: *ARNTL*), or *NFIL3* transcript level (Supplementary Fig. 1). As expected, gene expression of fibrotic markers, such as *COL1A1*, *COL1A2*, *COL3A1*, *COL5A2*, *TGFB1*, *TGFB2*, *VIM1*, *FN1*, and *MMP2* was increased at 14 days post bleomycin injury (Fig. 2a, b and Supplementary Fig. 1). Decreased expression of gene levels of *OCLN*, *TJP1*, *TJP3*, and *CDH1* were also observed, while *SMAD2* and *TJP2* showed no significant changes in the bleomycin group compared with PBS control (Fig. 2a, b and Supplementary Fig. 1). Similarly, we also observed the decreased protein expression of REV-ERBα in the bleomycin-treated group, as well as increased protein levels of LOX (total and activated) and COL1A1 (Fig. 2c).

### Exacerbated fibrotic progression and lung injury induced by bleomycin dosed at night

**Fig. 3:**
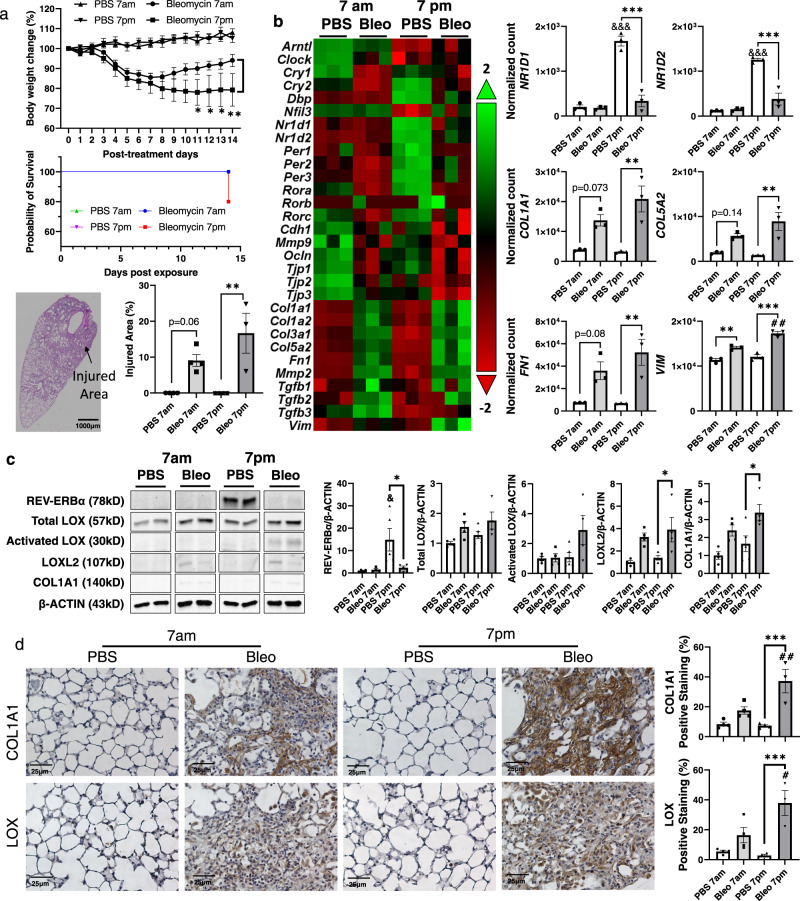
The health status of mice, circadian genes and fibrotic genes and protein expressions were affected by bleomycin injury in different time points (7 a.m. vs. 7 p.m.). C57BL/6J WT female mice were used for testing. **a** The body weights and survival rate were monitored until day 14 post-injury. (*n* = 3–5 mice per group, *p < 0.05, **p < 0.01 vs. bleomycin 7 a.m. group). Lungs were harvested, and H&E staining was performed to identify the injured area percentage. **b** RNA was isolated from lungs homogenates, and gene expression analysis was conducted using customized nanostring panel through nCounter SPRINT Profiler, and transcripts levels were normalized and visualized by nSolver software. The dysregulated genes were shown as a heatmap with circadian and profibrotic genes. Selected gene expressions were shown as bar graph (*n* = 3 mice per group). **c** Proteins isolated from lung homogenates were tested via western blot (REV-ERBα, LOX, Activated LOX, LOXL2, and COL1A1) represented blots were showing and change fold was normalized to β-ACTIN (*n* = 4–5 mice per group). **d** Lung sections were used for IHC, and the abundance and localization of COL1A1 and LOX were detected (*n* = 3–4 mice per group). Data were shown as mean ± SEM, two-way ANOVA followed Tukey’s multiple comparisons test was performed in **a** (Body weight change (%)) and one-way ANOVA followed Šídák’s multiple comparisons test was used in **a** (injured area (%); and **b**–**d**). Bar size: 1000 µm in **a**, and 25 µm in **d**. (*p < 0.05, **p < 0.01, ***p < 0.001 between groups; ^##^*p* < 0.01 vs. Bleo 7 a.m. group; ^&&&^*p* < 0.001 vs. PBS 7 a.m. group).

Since REV-ERBα expression occurs in circadian oscillation, the expression of REV-ERBα starts to increase from 6 a.m. (ZT0) and starts to decrease at 6 p.m. (ZT12)^19^. Thus, we dosed the mice at the beginning of the day (lights on) and night (lights off) cycles to determine whether the oscillation of REV-ERBα expression affects the fibrotic progression induced by bleomycin injury (Fig. 3). Interestingly, we found that mice treated with bleomycin at 7 p.m. exhibited exacerbated body weight loss compared to those dosed at 7 a.m. from days 11–14 post-injury (Fig. 3a). In addition, mice dosed at 7 a.m. had a 100% survival rate, whereas only 75% of the mice dosed at 7 p.m. survived (Fig. 3a). We also noticed that mice dosed with bleomycin at both 7 a.m. and 7 p.m. showed dramatic lung injury, and mice dosed at 7 p.m. showed more injury area in lung sections compared to mice dosed at 7 a.m. (Fig. 3a and Supplementary Fig. 2a).

We also investigated the gene expression from mouse lungs dosed with bleomycin at different times of day (Fig. 3b). Interestingly, the gene expression of *REV-ERBα/β* (*NR1D1*/*NR1D2*) was decreased after bleomycin injury during the night (dusk), whereas no significant difference was observed when dosed during the day (dawn) (Fig. 3b). The other circadian genes inhibited by *REV-ERBα/β*, such as *BMAL1* (*ARNTL*), and *CLOCK*, were differentially decreased during the day and had no change during the night (Supplementary Fig. 2b, c). The REV-ERBα/β competitor *RORA* showed decreased expression levels after bleomycin injury in either daytime or nighttime. We also observed the decreased gene expression levels of *PER1/2* and *CRY1/2* either bleomycin was dosed during the daytime or nighttime (Supplementary Fig. 2b, c). The gene expression of fibrotic markers, such as *COL1A1*, *COL5A2*, *FN1*, and *SERPINE1* was significantly upregulated when dosed at night (Fig. 3b and Supplementary Fig. 2b, c). Bleomycin injury increased *VIM* regardless of the time of dosing (Fig. 3b). The gene expression of *COL1A2* and *COL3A1* was upregulated after bleomycin dosing, and there was no time of a day difference during nighttime or daytime (Supplementary Fig. 2b, c). The gene expression of tight junction proteins responsible for cell-cell interaction: *TJP1* and *TJP3* showed significant downregulation when bleomycin was dosed at both day and nighttime, and *TJP3* showed further decreased during nighttime dosing (Supplementary Fig. 2b, c).

To further identify the expression level of target genes, we have detected the protein expression by western blot and IHC (Fig. 3c, d). Similarly, higher protein expression of REV-ERBα was observed in PBS 7 p.m. group compared to the PBS group at 7 a.m., and bleomycin injury significantly downregulated the protein abundance of REV-ERBα, whereas no changes in 7 a.m. groups (PBS vs. Bleo) (Fig. 3c). We also observed an increasing trend of protein level of total LOX after dosing bleomycin at either 7 a.m. or 7 p.m., while activated LOX only showed an increasing trend when bleomycin was dosed at 7 p.m. (Fig. 3c). The significantly increased protein abundances of LOXL2 and COL1A1 were observed in mice dosed with bleomycin at 7 p.m. while non-significant increase when dosed at 7 a.m. (Fig. 3c). Except for the western blot, we also detected the protein abundance and localization of LOX and COL1A1 via IHC (Fig. 3d). Bleomycin dosed at 7 p.m. showed higher protein levels of COL1A1 and LOX, especially in the injured area compared to mice dosed with bleomycin at 7 a.m. (Fig. 3d).

### Rev-erbα agonist attenuated the collagen overexpression during bleomycin-induced fibrogenesis

**Fig. 4:**
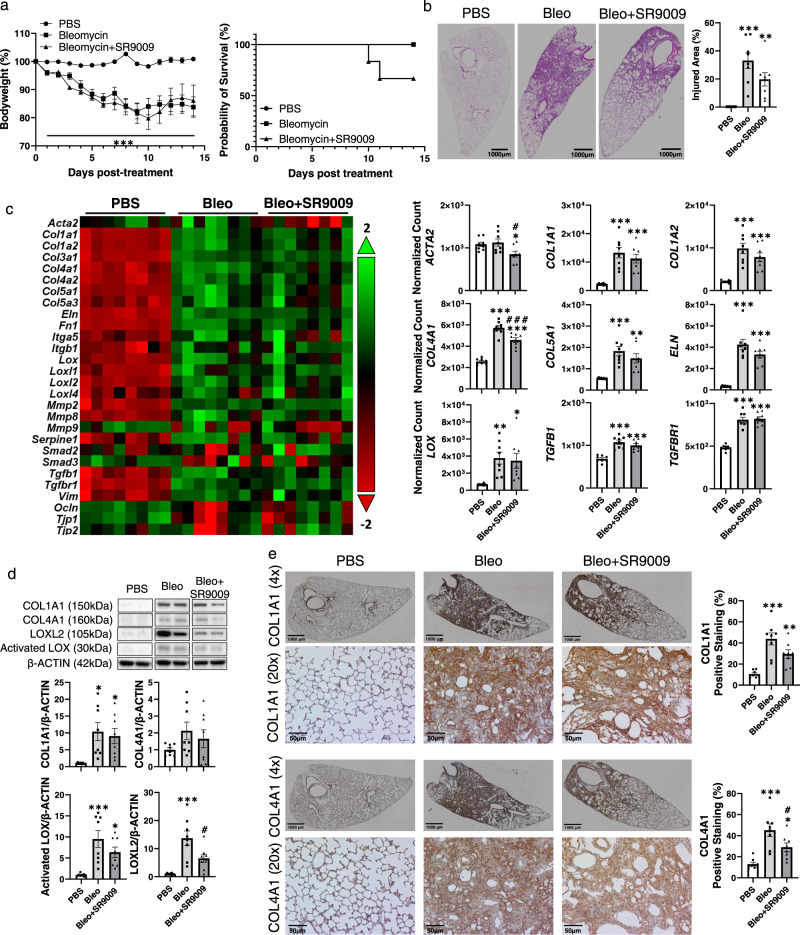
Rev-erbα agonist (SR9009) treatment helped to reduce the collagen overexpression occurred in bleomycin induced lung fibrosis. C57BL/6J WT mice (equal number of male and female mice) were dosed with bleomycin for 14 days, and SR9009 was given via i.p. injection at a dose of (100 mg/kg) daily. **a** The body weights and the survival rate was monitored until day 14 post-injury (*n* = 8–12 mice per group). **b** Lungs were harvested, and H&E staining was performed to identify the injured area percentage (*n* = 8 mice per group). **c** RNA was isolated, and gene expression analysis was conducted using nCounter Fibrosis panel via nCounter SPRINT Profiler, and transcripts levels were normalized and visualized by nSolver. The dysregulated genes focused on collagen dynamics and ECM remodeling were shown as a heatmap and selected gene expressions were shown as bar graphs (*n* = 8 mice per group). **d** Proteins isolated from lung homogenates were detected via western blot (COL1A1, COL4A1, LOXL2, and Activated LOX), represented blots were shown and fold change was normalized to β-ACTIN (*n* = 8 mice per group). **e** Lung sections were stained by COL1A1 and COL4A1 via IHC, and the abundance and localization were determined by ImageJ (*n* = 8 mice per group). Data were shown as mean ± SEM, multiple unpaired *t*-test was used for **a**, and one-way ANOVA followed Šídák’s multiple comparisons test was used in **b**–**d**. Bar size: 1000 µm in **b** and **e**, ×4 magnification, and 50 µm in **e**, ×20 magnification. (*p < 0.05, **p < 0.01, ***p < 0.001 vs. PBS group; ^#^*p* < 0.05, ^###^*p* < 0.001 vs. Bleo group).

Decreased abundance of REV-ERBα has been noticed after bleomycin injury, we treated mice with Rev-erbα agonist (SR9009, 100 mg/kg, intraperitoneally (i.p.)) for 14 days to determine it’s protective potential against fibrotic progression (Fig. 4 and Table 1). During 14 days post bleomycin injury, there was a significant reduction in body weight starting from day 1, and there is no significant difference between bleomycin and bleomycin + SR9009 groups (Fig. 4a). Surprisingly, only a 60% survival rate was observed in mice that received bleomycin + SR9009 while there was no death in the bleomycin-treated group (Fig. 4a). From the H&E stained sections, we identified that bleomycin-induced significant lung injury, and SR9009 treatment helped alleviate the injury but without significant difference (Fig. 4b). Since we were interested in how REV-ERBα is involved in pro-fibrotic progression, we measured the gene and protein expressions of fibrotic markers in the lungs (Fig. 4c–e and Supplementary Fig. 3). Although the *ACTA2* gene level was not significantly increased after bleomycin injury, the bleomycin + SR9009 group showed a significantly reduced expression of the *ACTA2* gene (Fig. 4c). We have noticed the significant upregulation of collagens (*COL1A1, COL1A2, COL3A1, COL4A1, COL4A2, COL5A1*, and *COL5A3*), and SR9009 treatment helped to reduce the levels of *COL1A1*, *COL1A2*, and *COL5A1* without significant difference, and the gene level of *COL4A1* was significantly downregulated after SR9009 treatment (Fig. 4c). Gene expression of lysyl oxidases (*LOX*, *LOXL1*, *LOXL2*, and *LOXL4*) were significantly increased after bleomycin injury, but SR9009 treatment did not help reduce the gene abundances (Fig. 4c and Supplementary Fig. 3). Other ECM proteins, such as *ELN* and *FN1*, were upregulated after bleomycin injury and SR9009 treatment helped to lower the transcript level but without a significant difference (Fig. 4c and Supplementary Fig. 3). As potential regulators of ECM remodeling and dysregulated repair, there were upregulated gene levels of *TGFB1*, *TGFBR1*, and *TGFBR2* after bleomycin injury, but no difference was observed between bleomycin and bleomycin+SR9009 treatment groups (Fig. 4c and Supplementary Fig. 3). Based on the gene expression results, we have performed a pathway analysis. Most significantly, ECM degradation, collagen biosynthesis and modification, and ECM synthesis were activated after bleomycin injury and slightly inhibited by SR9009 treatment (Table 1). Hence, we focused on how protein levels of collagen were affected.

**Table 1:** Dysregulated pathways after Bleomycin injury with or without SR9009 in C57 mice

Term ID	Directed enrichment score	
	Bleo vs. PBS	Bleo + SR9009 vs. PBS
ECM degradation	10.0612	9.6061
Collagen biosynthesis and modification	9.5866	9.1477
ECM synthesis	8.5745	7.81
PDGF signaling	7.0117	6.5104
SASP	6.197	5.7895
Myofibroblast regulation	4.2147	3.9987
TGF-beta	3.6261	3.132
M1 activation	3.4494	3.0848
De novo lipogenesis	2.5027	3.223
M2 activation	1.8004	0.8803
mTOR	1.6895	−0.3849
PPAR signaling	−1.6652	0.3668

Since we have observed the decreased transcript levels of multiple collagens in bleomycin + SR9009 group compared to bleomycin group, we tested the protein abundances of COL1A1, COL4A1, LOX, and LOXL2 as well (Fig. 4d, e). Similarly, we have observed increased protein levels of COL1A1 and COL4A1, and non-significant decreased trends of COL1A1 and COL4A1 after SR9009 injection (Fig. 4d, e). Interestingly, we have noticed a decreased trend of activated LOX, and a significantly decreased level of LOXL2 in the bleomycin + SR9009 group compared to the bleomycin group (Fig. 4d). From IHC staining, we observed overexpression protein levels of COL1A1 and COL4A1 in the injured sections from both the bleomycin group and the bleomycin + SR9009 group. However, the positive distribution of COL1A1 and COL4A1 were inhibited by SR9009 injection, and abundances of both collagens were slightly decreased in the bleomycin + SR9009 group compared to bleomycin group (Fig. 4e). There was no significant sex-dependent difference between bleomycin group and bleomycin + SR9009 group, hence we have combined male and female mice for further analysis.

### Rev-erbα deficiency exaggerated IAV-induced lung injury

**Fig. 5:**
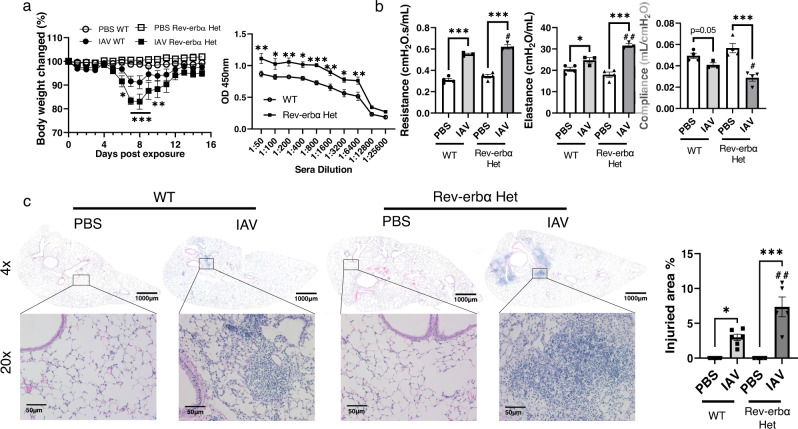
IAV induced lung injury and profibrotic responses exaggerated in Rev-erbα Het mice compared to WT mice. WT and Rev-erbα Het mice were infected (10^3^ PFU/mouse) with IAV or PBS control for 15 days. **a** Body weights were monitored during infection, and virus-specific antibodies in serum were detected by ELISA (*n* = 5–19 mice per group, *p < 0.05, **p < 0.01, ***p < 0.01 vs. IAV infected WT mice). **b** During sacrifice, lung mechanics (resistance, compliance, and elastance) were measured. (*n* = 3–4 mice per group). **c** H&E stained lung sections were used to analyze the injured area induced by IAV infection. Regions within the black squares were shown with ×20 magnification (*n* = 4–6 mice per group). Data were shown as mean ± SEM, two-way ANOVA followed Tukey’s multiple comparisons test was performed in **a** (bodyweight change (%)), multiple unpaired *t*-test was used for **a** (virus-specific antibodies titer), one-way ANOVA followed Šídák’s multiple comparisons test was used in **b**, **c**, unpaired two-side *t*-test was used in **b** (Resistance IAV-WT vs. IAV Rev-erbα Het; Elastance PBS-WT vs. IAV-WT; Compliance PSB-WT vs. IAV-WT and IAV-WT vs. IAV Rev-erbα Het). Bar size: 1000 µm in **c** (×4 magnification), and 50 µm in **c** (×20 magnification) (*p < 0.05, **p < 0.01, ***p < 0.001 between groups; ^#^*p* < 0.05, ^##^*p* < 0.01 vs. IAV infected WT mice).

To directly understand the role of Rev-erbα in pulmonary fibrogenesis and lung injury, we infected WT and Rev-erbα Het mice with IAV (10^3^ PFU) for 15 days to induce lung injury and fibrotic responses (Fig. 5). At 6–10 days post infection (p.i.), we found that IAV-induced weight loss was exacerbated in Rev-erbα Het mice compared with WT (Fig. 5a). We also monitored locomotor activity after IAV infection, and observed reduced ambulatory counts during the nighttime at 5 days to 9 days p.i. The locomotor activity showed no change during the daytime, and there was no significant difference between WT and Rev-erbα Het mice (Supplementary Fig. 4). After 15 days p.i., we collected the serum to detect IAV-specific antibody (IgG2a and IgA) levels. Both WT and Rev-erbα Het mice infected with IAV showed detectable levels of IgG2a and IgA in serum, and Rev-erbα Het mice showed higher levels of IgG2a and IgA compared to WT mice (Fig. 5a), most likely because of the highest level of infection. We also looked at the viral replication on 2 days and 4 days p.i. There was a significant increase in the viral titer at 4 days p.i., compared to 2 days p.i., while there was no significant difference in viral harboring and replication in the lungs between WT and Rev-erbα Het mice (Supplementary Fig. 5).

Furthermore, we determined the lung mechanical properties (airway resistance, elastance, and compliance). We have observed increased resistance and elastance, as well as decreased compliance after IAV infection compared with PBS control, both in WT and Rev-erbα Het mice. Intriguingly, the Rev-erbα Het mice exhibited increased resistance, elastance, and decreased compliance compared with WT mice in response to IAV infection (Fig. 5b). The H&E-stained lung sections showed IAV infection-induced dramatic lung injury with scaring progression in the alveoli of both WT and Rev-erbα Het mice. More importantly, larger injury areas were observed in Rev-erbα Het mice infected with IAV compared with IAV infected WT mice (Fig. 5c).

### Rev-erbα deficiency aggravated dysregulated gene expression during IAV-induced fibrogenesis

**Fig. 6:**
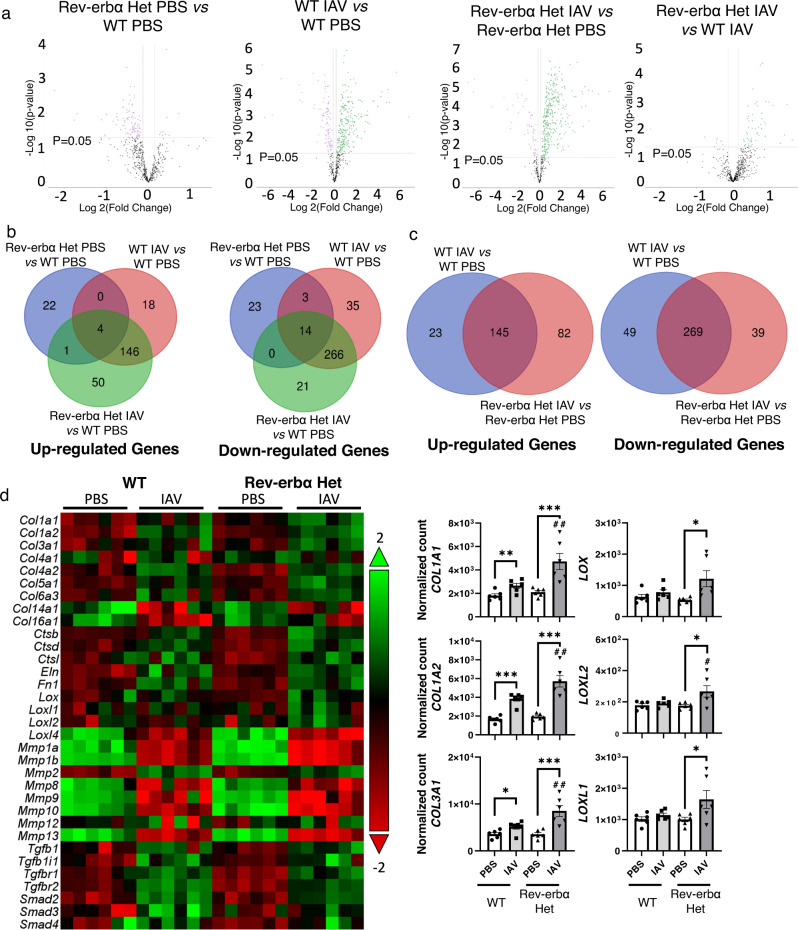
IAV infection induced dysregulation of profibrotic gene expression exacerbated in Rev-erbα Het mice. WT and Rev-erbα Het mice (equal number of male and female mice) were dosed with IAV (10^3^ PFU) for 15 days, and lungs were homogenized for RNA isolation. Gene expression analysis was conducted using nCounter Fibrosis Panel via nCounter SPRINT Profiler. RNA expressions were normalized and analyzed via nSolver software and ROSALIND service. **a** The dysregulated gene expressions between groups were shown as volcano plots, the cut off filter is at least 10% change (up or downregulation), and *p* < 0.05. **b**, **c** Overlapping gene expression changes among groups were shown by Venn diagrams with the same cutoff line used for volcano plots. **d** The overview of gene expression focused on collagen dynamics were shown as a heatmap, and the selected gene transcript levels (collagens and lysyl oxidases) were shown as a bar graph separately. Data are shown as mean ± SEM, one-way ANOVA followed Šídák’s multiple comparisons test was used in **d**, unpaired two-side *t*-test was used in **d** (*COL1A1* PBS-WT vs. IAV-WT and *COL3A1* PBS-WT vs. IAV-WT). (*n* = 6 mice per group; *p < 0.05, **p < 0.01, ***p < 0.001 between groups; ^##^*p* < 0.01 compared with IAV infected WT group).

**Fig. 7:**
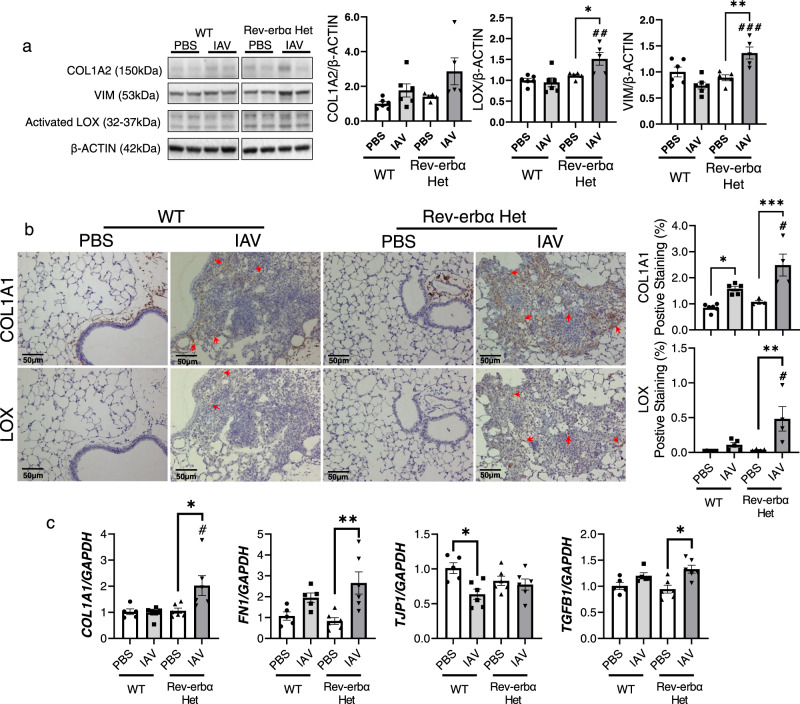
IAV infection induced dysregulation of profibrotic progression exacerbated in Rev-erbα Het mice. WT and Rev-erbα Het mice (equal number of male and female) infected (10^3^ PFU/mouse) with IAV for 15 days, and lungs were separated for RNA/protein isolation, or fixed with 10% formalin for FFPE sections. **a** The protein abundance of COL1A2, VIM and activated LOX were measured by western blot. Representative blot images were shown. Different targets were run on the same membrane: COL1A2, VIM and activated LOX were probed in the same membrane and β-ACTIN was used as an endogenous control (*n* = 5–6 mice per group). **b** The localizations of COL1A1 and LOX were determined by immunohistochemical staining, and red arrows were used to indicate the regions of interest. The positive staining area was calculated via ImageJ (*n* = 4–6 mice per group). **c** RNA isolated from lung homogenates was used to measure the gene expression (*COL1A1*, *FN1*, *TJP1* and *TGFB1*) via qRT-PCR, and *GAPDH* was used as an endogenous gene for normalization (*n* = 5–6 mice per group). Data are shown as mean ± SEM, one-way ANOVA followed Šídák’s multiple comparisons test was used in **a**–**c**. Bar size: 50 µm in **b**. (*n* = 4–6; *p < 0.05, **p < 0.01, ***p < 0.001 between groups; ^#^*p* < 0.05, ^##^*p* < 0.01 compared with IAV infected WT group).

After sacrificing the mice at 15 days p.i., we collected the lung tissues for RNA expression analysis (Figs. 6 and 7). At the gene transcript level, the most significantly dysregulated genes due to Rev-erbα knockdown were downregulated. There are a significant number of genes that were dysregulated when infected with IAV in both WT and Rev-erbα Het mice. Intriguingly, IAV infection in Rev-erbα Het mice led to an upregulation of significantly dysregulated gene transcripts compared to WT mice infected with IAV, which suggests that the altered gene expressions were not due to genotype differences (most dysregulated genes are decreased), but that Rev-erbα affects the specific gene expression during lung injury induced by IAV (Fig. 6a). Following the cutoff filters (10% fold change with *p* value < 0.05) used for volcano plots, we also analyzed the gene cluster via Venn diagrams analysis. Compared to WT mice treated with PBS, a total of 67 genes were dysregulated because of the genotype difference (vs. Rev-erbα PBS group) (Fig. 6b). In WT mice infected with IAV, 486 genes were significantly altered, and 430 genes were similarly altered in both WT and Rev-erbα Het mice. Intriguingly, 71 genes started to show a significant difference in Rev-erbα Het mice infected with IAV with no change in WT mice, and 56 genes showed a significant difference in WT mice with no change in Rev-erbα Het mice infected with IAV (Fig. 6b). By comparing IAV vs. PBS in the same genotype (WT IAV vs. WT PBS, and Rev-erbα Het IAV vs. Rev-erbα Het PBS), a total of 414 genes were commonly dysregulated when infected with IAV (Fig. 6c). Specifically, 121 genes were statistically dysregulated in Rev-erbα Het mice infected with IAV, and 72 genes showed a significant difference only in WT groups (PBS vs. IAV). The detailed gene lists corresponding to each comparison are listed in Supplementary Data 1. Based on the dysregulated gene lists, we identified pathways modified by IAV and Rev-erbα, which included collagen dynamics, EMT, TGFβ, myofibroblast regulation, as well as M1/M2 macrophage activation. These pathways were upregulated after IAV infection and were further exacerbated when Rev-erbα was diminished (Table 2). Additionally, collagen biosynthesis and modification, ECM degradation, and ECM synthesis were among the most upregulated pathways in Rev-erbα Het IAV-infected mice compared to IAV-infected WT mice (Table 2). Hence, we focused our study on the alteration of specific genes/proteins related to collagen biosynthesis, modification, and degradation.

**Table 2:** Dysregulated pathways after IAV infection in both WT and Rev-erbα Het mice

Term ID	Directed enrichment score			
	Rev-erbα Het PBS vs. WT PBS	WT IAV vs. WT PBS	Rev-erbα Het IAV vs. Rev-erbα Het PBS	Rev-erbα Het IAV vs. WT IAV
Collagen biosynthesis and modification	−0.73	1.5086	4.8161	2.6449
De novo lipogenesis	−1.1365	1.609	2.4369	0.811
ECM degradation	−0.7221	1.2625	4.1753	2.1627
ECM synthesis	−0.5674	1.9623	4.1307	2.2267
EMT	−1.1894	2.0273	3.7485	1.2953
Focal adhesion kinase	−1.0713	1.0623	3.3423	1.4581
Inflammasome	−1.3059	1.5815	2.9338	0.4385
M1 activation	−1.1324	1.5827	3.1153	0.7314
M2 activation	−0.2606	−1.0765	1.7617	0.6458
Myofibroblast regulation	−1.2826	2.2823	4.0281	1.3925
TGFβ	−1.2466	0.9978	3.1502	1.3947
Wnt	−1.6523	1.262	3.2124	0.2816

### Absence of Rev-erbα exacerbated the activated collagen stabilization and modification during IAV-induced fibrogenesis

To further determine the role of Rev-erbα on collagen dynamics, we measured gene expression related to collagen modification, ECM markers, matrix metalloproteinases (MMPs), and TGFβ pathways (Fig. 6d and Supplementary Fig. 6). We noticed that collagens were significantly upregulated after IAV infection in both WT and Rev-erbα Het mice at the gene expression level. In particular, *COL1A1*, *COL1A2*, *COL3A1*, and *COL5A1*, were significantly increased in IAV-infected Rev-erbα Het mice compared with the WT IAV group (Fig. 6d and Supplementary Fig. 6a). Interestingly, we observed decreased *COL14A1* and *COL16A1* in IAV-infected mice, but there was no difference between the WT IAV group and Rev-erbα Het IAV group (Fig. 6d). We also noticed that lysyl oxidases (*LOX*, *LOXL1*, and *LOXL2*) were upregulated only in IAV-infected Rev-erbα Het compared to PBS-treated Rev-erbα Het mice, and there were no significant difference between IAV infected and PBS-treated WT mice (Fig. 6d). In addition, we noticed the upregulation of other ECM-related genes, such as *FN1*, *ELN*, *VIM*, *ITGA4*, *ITGA9*, and *HSPG2*, and genes responsible for focal adhesion such as *LAMA3* and *OCLN* were downregulated after IAV infection in either genotype, and there is no significant difference between IAV infected WT and Rev-erbα Het mice (Fig. 6d and Supplementary Fig. 6a). One of the key genetic pathways activated during fibrotic progression is the TGFβ pathway, and we found increased activation of TGFβ signaling following IAV infection, which showed increased *TGFB1*, *TGFB1I1*, *TGFBR1 TGFBR2*, *SMAD2*, *SMAD3*, and *SMAD4*. However, there was no significant difference between the IAV WT and the IAV Rev-erbα Het groups (Supplementary Fig. 6b). Since we observed increased collagen abundance, we also determined the expression of related collagenases, MMPs (Supplementary Fig. 6c). Increased gene expression of *MMP2*, *MMP12*, *MMP14*, and *MMP12* was observed only in Rev-erbα Het mice infected with IAV compared with PBS in the same genotype. The gene transcript levels of *MMP2* and *MMP14* showed a significant increase in Rev-erbα Het mice infected with IAV compared to WT mice infected with IAV (Supplementary Fig. 6c). Other MMPs, such as *MMP9*, *MMP8*, and *MMP3*, showed decreased gene transcript levels when IAV infection occurred, and there is no difference between the two genotypes. The inhibitor of MMPs: TIMPs, showed increased *TIMP2* in Rev-erbα Het IAV group compared to Rev-erbα Het PBS group (Supplementary Fig. 6c).

### Lack of Rev-erbα augments collagen overexpression during IAV-induced fibrogenesis

Since we observed that type 1 collagen and lysyl oxidases were upregulated in gene transcript levels, we also tested the protein abundance and localization (Fig. 7). Overall, from lung homogenates, we found an increasing trend of type 1 collagen (COL1A2) without statistical significance. Meanwhile, we noticed a significant increase in LOX and VIM in Rev-erbα Het mice infected with IAV compared to either the Rev-erbα Het PBS group or the WT mice infected with IAV (Fig. 7a). Further, we looked at the protein abundance and localization of COL1A1 and LOX. We performed IHC staining of COL1A1 and LOX (Fig. 7b). The distribution of COL1A1 in PBS treated group, either WT or Rev-erbα Het mice, was around the small airways or bronchial. When IAV infection occurred, COL1A1 was augmented in the injured area, mainly around the alveoli. Furthermore, no COL1A1 was observed in alveoli in the PBS-treated group (Fig. 7b). However, for the protein expression of LOX, relatively lower level of LOX were observed in IAV-infected WT mice, while the abundance of LOX were increased in Rev-erbα Het mice infected with IAV, primarily localized to the injured area (Fig. 7b). Since lysyl oxidase is responsible for collagen stabilization via crosslinking the collagen fibers, the co-localization of LOX and collagen in areas of injury was observed as expected (Fig. 7b, red arrow). We also applied the qRT-PCR to detect the gene expression fold change, and we noticed a similar trend compared with NanoString analysis (Fig. 7c). The gene transcript level of *COL1A1*, *FN1*, and *TGFB1* showed an increasing trend after IAV infection, and Rev-erbα knockdown exacerbated the upregulation. The gene expression of *TJP1* was decreased in the WT group with IAV infection (Fig. 7c).

### Rev-erbα agonist attenuated the TGFβ-induced abnormal collagen stabilization and fibrotic responses in lung fibroblasts

**Fig. 8:**
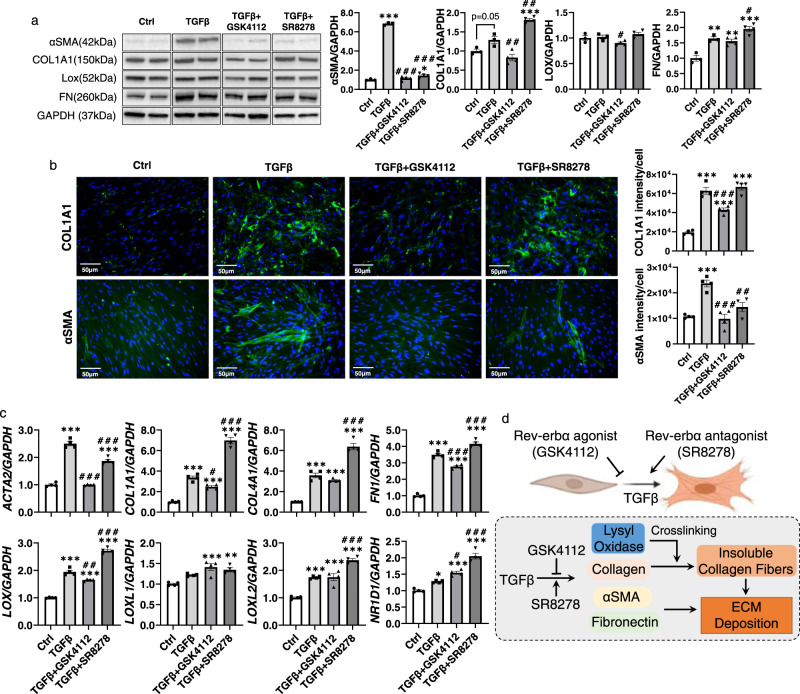
Rev-erbα agonist inhibits TGFβ induced fibroblast differentiation and antagonists exacerbate it. Human primary lung fibroblast were treated with TGFβ (2 ng/ml) with or without Rev-erbα agonist (GSK4112, 20 µM) or antagonist (SR8278, 20 µM) for 2 days. **a** Protein was isolated for western blot analysis (αSMA, COL1A1, LOX, and Fibronectin (FN)). Represented blots are shown with densitometry analysis (*n* = 3–4 cells per group). **b** Immunofluorescence staining showed the distribution and protein abundance of COL1A1 and αSMA, DAPI was used for nuclear staining (×20). Relative fluorescence intensity was calculated in ImageJ, as fluorescence intensity per cell (*n* = 4 cells per group). **c** RNA was isolated for gene expression measurement via qPCR (*ACTA2*, *COL1A1*, *COL4A1, FN1, LOX*, *LOXL1, LOXL2*, and *NR1D1*). GAPDH was used as an endogenous control for RNA and protein fold change normalization (*n* = 4 cells per group). Data are shown as mean ± SEM, one-way ANOVA followed Šídák’s multiple comparisons test was used in **a**–**c**, unpaired two-side *t*-test was used in **a** (COL1A1 Ctrl vs. TGFβ, LOX TGFβ vs. TGFβ + GSK4112, FN TGFβ vs. TGFβ + SR8278). **d** Schematic demonstrating how both Rev-erbα agonist and antagonist regulates ECM deposition in lung fibroblast induced by TGFβ, and the schematic is created with Biorender.com. Bar size: 50 µm in **b**. (*p < 0.05, **p < 0.01, ***p < 0.001 vs. Ctrl group; ^#^*p* < 0.05, ^###^*p* < 0.001 vs. TGFβ group).

To determine the role of Rev-erbα in the abnormal collagen modification via lysyl oxidase, we treated primary adult human lung fibroblasts (HLF) and human fetal lung fibroblasts (HFL1) with TGFβ (2 ng/ml) with or without Rev-erbα agonist (GSK4112, 20 μM) and antagonist (SR8278, 20 μM) for 2 days (Fig. 8 and Supplementary Figs. 7 and 8).

We previously found that Rev-erbα agonist, GSK4112, inhibited the myofibroblast differentiation induced by TGFβ^27^. Here we noticed that TGFβ induced myofibroblast differentiation was inhibited by GSK4112, while exacerbated by SR8278 (Fig. 8). Significantly, GSK4112 inhibited the TGFβ induced overexpression of αSMA and COL1A1 in protein levels (Fig. 8a, b), and TGFβ upregulated gene levels of *ACTA2*, *COL1A1*, *FN1*, and *LOX* were inhibited by GSK4112 treatment (Fig. 8c). In addition, treatment of SR8278 exacerbated the TGFβ upregulated COL1A1 and FN in protein levels (Fig. 8a), and augmented the TGFβ increased transcript levels of *COL1A1*, *COL4A1*, *FN1*, *LOX*, and *LOXL2* (Fig. 8c). Interestingly, we found that both GSK4112 and SR8278 increased the gene level of *NR1D1* (Fig. 8c). Based on our results, we concluded that Rev-erbα agonist helped to attenuate the TGFβ-induced fibroblast differentiation and collagen overexpression, while Rev-erbα antagonist exacerbated it (Fig. 8d).

We also tested our hypothesis in HFL1 (Human fetal lung fibroblast) (Supplementary Figs. 7 and 8). GSK4112 treatment showed a significantly increased gene transcript level of *NR1D1* while SR8278 showed no change in HFL1 (Supplementary Fig. 7). In addition, GSK4112 inhibited TGFβ*-*induced *ACTA2* and slightly decreased gene expression of *COL1A1* and *FN1* without significant difference in TGFβ + GSK4112 group compared to TGFβ treatment alone (Supplementary Fig. 7). Intriguingly, we also found that GSK4112 alleviated the upregulated gene expression of lysyl oxidases (*LOX*, *LOXL1*, and *LOXL2*) (Supplementary Fig. 7). We also measured the protein abundance of COL1A1 and LOX. Similarly, GSK4112 suppressed the upregulated protein level of LOX induced by TGFβ. In contrast to the gene expression results, TGFβ-induced COL1A1 protein was significantly inhibited by GSK4112 treatment, and the protein fibers overexpressed by TGFβ were also significantly repressed by GSK4112 (Supplementary Fig. 8). Treatment with Rev-erbα antagonist (SR8278) showed no significant effects on TGFβ-induced fibroblast differentiation or collagen stabilization in HLF1 (Supplementary Fig. 8).

### Rev-erbα agonist and antagonist exacerbated the TGFβ-induced epithelial-mesenchymal transition (EMT) in lung epithelium

We have also treated primary human small airway epithelial cells (SAEC) and human bronchial epithelial cell line (BEAS-2B) with TGFβ (2 ng/ml) with or without Rev-erbα agonist (GSK4112, 20 μM) and antagonist (SR8278, 20 μM) for 2 days (Supplementary Figs. 9 and 10). SAEC treated with TGFβ showed activated EMT tendency via increased *VIM*, *LOXL2*, and *COL1A1*, as well as decreased *CDH1*, *TJP1*, and *OCLN*. The treatment of GSK4112 and SR8278 showed exacerbated gene dysregulation of both epithelial and mesenchymal markers (Supplementary Fig. 9a). Protein levels of COL1A1 and VIM were upregulated by TGFβ and the upregulation was prevented by GSK4112 (Supplementary Fig. 9b). Similar to HLF, both agonist and antagonist treatment showed increased gene expression of *NR1D1*.

In BEAS-2B, increased gene levels of *COL1A1* and *FN1* were noticed after TGFβ treatment, and GSK4112 attenuated the upregulation, whereas SR8278 exacerbated the gene levels of *FN1* with significance and *COL1A1* without significance (Supplementary Fig. 10). A significantly increased *LOX* gene level was observed after GSK4112 or SR8278 treatment compared to the TGFβ group. Either GSK4112 or SR8278 inhibited increased *LOXL1* by TGFβ treatment. TGFβ inhibited the gene expressions of *LOXL2* and *ACTA2*, and SR8278 treatment eliminated the downregulation, but there was no effect with GSK4112 treatment (Supplementary Fig. 10).

## Discussion

Pulmonary fibrosis is a lethal chronic lung disease without effective therapeutic options, and the pathogenesis of fibrogenesis remains unclear^6,33,34^. Recent studies have demonstrated the novel role of the circadian molecular clock in the pathobiology of chronic lung diseases and highlighted the potential for circadian clock-based therapeutics^23,35^. Targeting specific circadian clock genes has been implicated with anti-fibrotic potential in vitro in cells or in vivo in mouse models of lung injury^8,36–38^. In previous studies, Rev-erbα deficiency exacerbated the EMT induced by CS and fibrogenesis induced by bleomycin, and Rev-erbα agonist inhibited the fibroblast differentiation induced by TGFβ^8,27^. In this study, we have characterized the Rev-erbα abundance in human IPF patients histologically, as well as in a bleomycin mouse model, and we found the decreased REV-ERBα protein abundance, especially in IPF lesion areas and bleomycin-induced fibrogenesis. Based on our results, the lower protein abundance of REV-ERBα in the healthy portion of IPF patients could promote the progression of fibrogenesis toward a lesion phenotype. Since the abundance of REV-ERBα is in circadian oscillation, mice dosed with bleomycin when REV-ERBα expression naturally starts to decrease (dark phase/nighttime) exhibited higher mortality and exacerbated fibrotic progression compared with those dosed in the daytime. We have administrated the Rev-erbα agonist (SR9009) to mice treated with bleomycin, and we noticed that SR9009 injection helped ease the collagen overexpression during bleomycin-induced fibrogenesis. We also analyzed whether diminished REV-ERBα exacerbated the fibrotic progression induced by IAV infection. Our results show that Rev-erbα regulated collagen stabilization via lysyl oxidase, and its agonist prevented TGFβ induced overexpression of collagen.

Circadian clock molecules RORα (nuclear receptor), REV-ERBα, BMAL1, and CLOCK, have been implicated in the crosstalk between inflammation and lung tissue injuries^19,22,39,40^. The critical circadian molecules: BMAL1 and CLOCK form a heterodimer that binds to E-Box and subsequently promotes the expression of Rev-erbα. Rev-erbα binds to RORE to prevent the expression of BMAL1 and CLOCK, while RORα activates the expression of BMAL1 and CLOCK. Both RORE and E-box are associated with EMT^41,42^, which is initiated at the early stage of fibrosis. Hence, the regulators of RORE and E-box (RORα, REV-ERBα, BMAL1, and CLOCK) are equally critical in fibrogenesis. In our results, we noticed that the gene level of BMAL1 (*ARNTL*) in the bleomycin model depended on the time of dosing. Decreased *BMAL1* was observed when dosed during the day, while an increasing trend of *BMAL1* transcript level was observed when treatment occurred during the nighttime. Similar time-dependent changes also occurred with *CLOCK* expression. Upregulated *BMAL1* was identified in fibrotic mouse lungs induced by TGFβ transfection, and *BMAL1* silencing helped to inhibit the fibrotic progression induced by TGFβ in the lung epithelium^36^. In the same report, TGFβ transfected into mouse lungs decreased the gene levels of *REV-ERBα*. REV-ERBα was also shown to be inhibited during fibrotic progression^36^. These published results agree with our data that show decreased REV-ERBα and increased *BMAL1* expression in bleomycin-induced fibrosis. Bleomycin-induced downregulation of REV-ERBα and increased *BMAL1* during the night might be one of the reasons for fibrotic progression and exacerbation. Interestingly, the gene expression of *CLOCK* showed very similar results to *BMAL1* gene alterations. It is known that *CLOCK* disruption exacerbates fibrotic progression, which partially agrees with our data on night dosing that *CLOCK* level showed lower expression during the night^37^. In the same study, bleomycin dosing at night showed more collagen deposition in the injured area, which supports our results^37^. Another study demonstrated that infection with IAV that occurred during the night showed worse body weight loss, higher mortality, and more severe tissue injury^22^. Our data also indicate the possibility that targeting BMAL1 or CLOCK as a potential candidate might need to consider the dosing time, and inhibition or activation of BMAL1 or CLOCK might be time-dependent.

Our results showed decreased REV-ERBα after bleomycin injury during the nighttime. The expression of REV-ERBα starts to decrease naturally during the night (dusk) and dosing with bleomycin-induced significantly decreased REV-ERBα levels could dampen the basal expression of REV-ERBα which might result in worse fibrotic phenotypes and health status. Since Rev-erbα is a key component of circadian molecular clock that shows a rhythmic expression^21^, i.e., the level of REV-ERBα starting to decrease from 6 p.m. could result in less protection against bleomycin-induced lung inflammation in mice dosed at ZT13, while increasing oscillation of REV-ERBα during the day could attenuate the inflammatory response caused by bleomycin dosed at ZT1. As mentioned before, dosing with bleomycin at night exacerbated the collagen deposition in the lungs, which agrees with our gene and protein expression results^37^. Surprisingly, we have observed the augmented protein expression of LOX in the lung injured area when dosed at 7 p.m. compared to the 7 a.m. group, and LOX is responsible for crosslinking collagen fibers to prevent the degradation of collagens^43,44^. To measure the REV-ERBα expressions in IPF patients, we stained for REV-ERBα in pulmonary fibrotic lesion areas. We observed a decreased abundance of REV-ERBα especially in the lesion area, while REV-ERBα was fully expressed in the healthy samples. Currently, limited studies directly report the expression levels of REV-ERBα (*NR1D1*) in IPF patients or bleomycin-induced fibrosis^45,46^. The single-cell RNA sequencing comparison between IPF and healthy patients identified the significant downregulation of *NR1D1* in ATII cells in IPF patients compared to healthy controls^45^. Our results showed significantly decreased REV-ERBα protein abundance, especially within the injured areas, which partially agrees with the previous study. Another study reported that the REV-ERBα mRNA level was decreased in bleomycin-induced lung fibrosis in young mice, as well as in the naturally aged mice lungs^46^. Our results show decreased REV-ERBα after bleomycin injury. Moreover, our study shows that bleomycin-induced downregulation of REV-ERBα occurs only during the nighttime. The level of REV-ERBα was unchanged when dosing in the daytime. Decreased REV-ERBα has been reported as a cause of exacerbation of fibrotic progress in either mouse or human lung fibroblasts^8^. Our results and previous publications suggest that REV-ERBα is inhibited during fibrogenesis and that decreased REV-ERBα either by transgenic methods or natural circadian oscillation exacerbates the fibrotic progression and worsens the lung injury.

We administered Rev-erbα agonist (SR9009) into mice dosed with bleomycin, and tested the protective effect of Rev-erbα agonist against fibrosis. We did not observe any difference in body weight decline between bleomycin alone and bleomycin with SR9009, however, we observed a lower survival rate in mice when received SR9009 post bleomycin injury. It has been proven that Rev-erbα agonist could increase body weight loss and fat mass loss^12^. Moreover, SR9009 has been shown to decrease cell viability and dysregulate cellular metabolism^47^. There are clinical reports describing that body weight loss and lower body mass index could worsen IPF progression and even lower the survival probability^48,49^. SR9009 accelerated body weight loss could be one of the reasons for the higher death rate in the bleomycin + SR9009 group compared to the bleomycin group. However, other side-effects of SR9009 might be contributing to the cause of death as well. More detailed studies should be conducted to understand the molecular mechanism of the off-target effects of SR9009 during fibrogenesis. Besides the side effects of SR9009, injection of the agonist helped inhibit the collagen contents at the gene and protein levels, which agrees with our results from the cell model. Our and other results showed that SR9009 had specificity in regulating the overexpression of collagen and helped to prevent fibrogenesis while the side effects need further investigation for the pre-clinical trial.

Previously, we have shown that Rev-erbα was associated with fibrotic responses during IAV infection in Rev-erbα Het mice, which led to fibrogenesis. After 15 days p.i., we noticed that Rev-erbα Het mice infected with IAV showed worse health status. The exaggerated upregulation of lung elastance was observed in Rev-erbα Het mice, demonstrating that Rev-erbα deficiency exacerbated the fibrotic progression functionally. To support our hypothesis, we have measured multiple fibrotic markers, such as type 1/3/5 collagens and lysyl oxidases (*LOX*, *LOXL1*, and *LOXL2*), which were only significantly upregulated by IAV in Rev-erbα Het mice. A previous study proved that Rev-erbα knockdown could exacerbate the fibrotic response by increasing αSMA protein expression^8^. Collagens are equally important in pulmonary fibrosis as αSMA, both of which are overexpressed during fibrogenesis and induce irreversible scarring. Our results elaborate on the previous reports and demonstrate that Rev-erbα is essential in regulating αSMA and correlated with collagen expression. As we mentioned before, Rev-erbα starts to decrease naturally during the nighttime, and it has been identified that IAV infection during the night is associated with worse health outcomes in mice, as well as higher mortality and more severe lung injury compared to daytime infection^22^. Another study reported that dosing bleomycin during the night increased collagen deposition compared to dosing during the day^37^, which also concurred with our findings here. Our data support previously published results and provide a possible explanation for why IAV infection at nighttime induces worse lung injury and higher mortality than during the day, when Rev-erbα starts to decrease. Our conclusion raises a possibility that working during the night shift could be more vulnerable to environmental hazards, which could contribute to developing fibrosis.

To understand the correlated signaling pathways involved with Rev-erbα in IAV infection-induced pulmonary fibrotic responses, we analyzed the directed enrichment scores to determine the related pathway. We noticed an exacerbated upregulation of multiple biological processes, such as collagen biosynthesis and modification, ECM degradation and synthesis, M2 macrophage activation, myofibroblast regulation, TGFβ pathway, and EMT. The most abnormal activation was in collagen synthesis and modification pathways, and we found the exaggerated upregulation of lysyl oxidases in Rev-erbα Het mice compared with WT mice infected with IAV. Both gene and protein expressions of lysyl oxidases were upregulated in Rev-erbα Het mice infected with IAV, but not in WT mice infected with IAV. Lysyl oxidases are known to stabilize the collagen fibers via crosslinking to prevent collagen degradation and improve tissue scarring^43,44^. Other than collagen, lysyl oxidases are also responsible for crosslinking elastin, which was further upregulated in Rev-erbα Het mice infected with IAV. Besides the collagen stabilization and synthesis, we also determined the expression of related collagenases (i.e., MMPs). We found the exacerbated increased *MMP2*, *MMP12*, and *MMP14* in Rev-erbα Het mice infected with IAV. The substrates of MMP2, MMP12, and MMP14 include gelatin, type 1 and 4 collagen, and elastin;^50^ Upregulated MMPs could be a self-regulating method for digesting the overexpressed ECM. Other MMPs, such as MMP9 and MMP8, which are responsible for digesting gelatin, collagen, and elastin, showed downregulation after IAV infection. The balance of MMPs as ECM regulators during fibrosis progress needs more detailed studies to understand how MMPs are involved in collagen dynamics, particularly during episodes of fibrogenesis.

Our previous study showed the therapeutic potential of Rev-erbα agonist in preventing EMT induced by cigarette smoke (CS) and fibroblast differentiation induced by TGFβ^27^. In this study, we found that Rev-erbα agonist treatment can prevent the abnormal collagen modification induced by TGFβ, and inhibits the overexpression of collagen. A previous study showed that Rev-erbα agonist could attenuate the fibrotic responses in vivo, ex vivo, and in vitro by measuring traditional fibrotic markers: *ACTA2* and *COL1A1*^8^. Our results further support the role of Rev-erbα in the fibrotic response. Rev-erbα agonist prevents the overexpression of collagen 1 and 4, lysyl oxidase, fibronectin, and αSMA, whereas Rev-erbα antagonist augments it. We found that Rev-erbα agonist treatment suppressed mRNA and protein expression of collagen caused by TGFβ with significance. Our results further described that Rev-erbα involvement in fibrotic progression might be through lysyl oxidase, which is known for stabilizing collagen content. It has been shown that SR8278 could promote myogenesis in myoblasts, but it has a very poor half-life: 0.17 h^51,52^. Similarly, we noticed that the Rev-erbα antagonist (SR8278) exacerbates the myofibroblast differentiation by augmenting the expression of collagen, lysyl oxidase, and fibronectin. Surprisingly, either Rev-erbα agonist or antagonist exacerbated the EMT induced by TGFβ in SAEC, while the cell-type specific role of Rev-erbα in vitro in lung cells needs further investigation. From our results, we showed a decreased Rev-erbα abundance during lung fibrogenesis, loss of Rev-erbα could exacerbate the fibrotic process induced by IAV infection via collagen-lysyl oxidase interaction, and pharmacological activation of Rev-erbα prevented the overexpression of collagens. Our and others studies demonstrate that mice dosed with bleomycin at night show worse fibrotic progress than during the day, which might be the result of decreasing Rev-erbα levels^37^. Based on our and others findings, circadian clock is critically involved in disease development, and night shift workers could face a higher chance of fibrotic disease development^22^, targeting the clock molecule Rev-erbα might be one potential therapeutic strategy to overcome the risk.

Current FDA-approved anti-fibrosis drugs: Nintedanib and Pirfenidone, are not targeting the lysyl oxidase mediating collagen stabilization^53,54^. Our findings suggest that Rev-erbα agonists possess great potential in protecting fibrogenesis by disrupting collagen fibers. Currently, there is a drug, PXS-5505: Pan-Lysyl Oxidase Inhibitor, that is in phase 1 clinical trials for myelofibrosis^55^. Rev-erbα agonists also preserve the possibility of treating pulmonary fibrosis, while the first generation of agonist: GSK4112 has poor pharmaceutical properties, and new Rev-erbα ligands are needed^12,51^. Based on the chemical structure of GSK4112, there are new agonists currently designed and available, such as SR9009, SR9011, GSK2945, SR12418, GSK2667, and GSK5072^12^. We have proven that SR9009 daily injection can prevent the EMT in lungs induced by 10 days of CS exposure^27^. Moreover, SR9009 attenuated liver fibrosis in mice with inhibited collagen expression^56^. Other agonists, such as SR12418, GSK5072, and GSK2667, have been identified that can inhibit inflammatory responses in THP1 cells^57,58^. Although there are disadvantages of Rev-erbα agonists in in vivo models, such as short half-life and off-target effects^47,51^, numerous reports including this study demonstrate the fundamental role of Rev-erbα in lung injury, and Rev-erbα agonists can prevent lung inflammation and injury induced by CS or IAV infection. A detailed study of the anti-fibrotic properties of different Rev-erbα agonists is needed to identify a proper agonist with suitable pharmaceutical characteristics for in vivo study, and even clinical trials.

Overall, Rev-erbα abundance was decreased in fibrotic progression, and naturally reduced Rev-erbα exacerbated the fibrogenesis. Rev-erbα deficiency exaggerated the fibrotic responses and lung injury induced by IAV infection, and Rev-erbα was involved in the activation of collagen stabilization via lysyl oxidase during the fibrotic progression caused by IAV. Treatment with Rev-erbα agonist can prevent the induction of collagen-lysyl oxidase interactions and stabilization. Our results support the fundamental role of Rev-erbα in fibrogenesis development. Rev-erbα agonists offer promising potential in preventing collagen overexpression and may help break down collagen fibers by inhibiting lysyl oxidase overexpression. Investigating other circadian clock molecules in fibrogenic progression might help us understand the molecular mechanism as well as discover novel therapeutic targets for treating pulmonary fibrosis.

## Methods

### Ethical approval

The experiments performed in this study were approved by the Animal Research Committee of the University of Rochester, University of Rochester Institutional Biosafety Committee, and the ethical standards from United States Animal Welfare Act and NIH.

### Human lung tissue slides declaration

Human lung samples (formalin fixed-paraffin embedded (FFPE) blocks, both Normal and IPF patients) were purchased from Origene (OriGene Technologies Inc). The detailed patient information and Sample/Label IDs are listed in Supplementary Table 1. Lung sections were prepared from the FFPE blocks with 5 μm thickness using a microtome. The sections are used for immunohistological chemistry (IHC) staining.

### Animals and treatments

Rev-erbα global heterozygous (Rev-erbα Het) mice (male and female mice, 2–4 months old) were purchased from Jackson laboratory (Strain #:018447), and adult C57BL/6 wild-type (WT, male and female mice, 2–4 months old) were bred in the vivarium at the University of Rochester Medical Center. Before treatment, mice were transferred to the inhalation core facility and allowed 1 week acclimatization period. The mice were housed on a 12/12 h light–dark cycle with ad libitum access to water and food. WT C57BL/6 mice were used for bleomycin dosing. Mice received 1.5 units/kg for 14 days, and bleomycin (Cat#1076308, Sigma) was delivered by oropharyngeal inhalation, after anesthetizing with isoflurane. During 14 days of dosing, Rev-erbα agonist (SR9009, Cat#554276, Sigma) was injected intraperitoneally (i.p.) between 11 a.m.–12 p.m. every day, at the dosage of 100 mg/kg body weight. SR9009 was prepared in 15% Kolliphor EL (Cat#: C5135, Sigma) as described previously^27^. The mice were sacrificed 14 days post-dosing, and the lungs were snap-frozen for further analysis. For IAV dosing, mice were anesthetized with isoflurane, and a total amount of 10^3^ plaque-forming units (PFU)/mouse of influenza A/Puerto Rico 8/1934 H1N1 virus (PR8) was given to mice intranasally^59^. A total of 3 female mice were housed individually ad libitum food and water were supplied in a special cage with a running wheel connected to an automatic counter. Mice were accommodated in the wheel running cage for 1 week, and counters were adjusted during this week. The locomotor activity was monitored from day 0 to day 14, and the mice were sacrificed on day 15 post-infection (p.i.). During 15 days of infection, body weights were monitored daily. A separate group of mice was placed in cages with the running wheel assembled, and the running wheel was connected to an automatic counter. Each cage has one mouse with access to regular water and food. The locomotor activity was recorded during 14 days of infection. During sacrifice, mice were anesthetized with pentobarbital (100 mg/kg) via i.p. injection. Lung function parameters (resistance, compliance, and elastance) were measured during the sacrifice via the Flexivent FX1 Legacy system (Scireq) following the manufacturer’s instructions. Each measurement was performed 3 times per animal. Mice lungs were also inflated with 1% low melting agarose and fixed with 10% formalin overnight for histological staining. Bleomycin and IAV dosing was regularly conducted between 11 a.m.–1 p.m. and sacrificed during a similar time. The time of day bleomycin dosing (7 a.m. and 7 p.m.) was performed in C57BL/6 female mice, and mice were sacrificed at the same time of day as their respective dosing. During 14 days, body weight was monitored. Another group of mice was dosed with an equal volume of PBS as the control group.

### Viral titer in lungs and IgG2a and IgA in serum measurement

Mice were sacrificed at 2 and 4 days p.i., lungs were collected and snap frozen for preparing the lung homogenates for viral titer measurement according to our previous publication^60^. Mice were sacrificed at 15 days p.i., and whole blood was collected through the posterior vena cava vein. Serum was separated from whole blood by centrifugation (12,000 × *g*, 10 min at room temperature). The IAV-specific IgG2a and IgA antibodies in serum were determined using ELISA via serial dilution as described in our previous publication^60^.

### Cell culture and treatment

Primary human lung fibroblast (Cat# CC-2512) and small airway epithelial cells (SAEC) (Cat# CC-2547) were purchased from Lonza. Lung fibroblasts were cultured in FGM-2 Fibroblast Growth Medium (Cat# CC-3132), and SAEC were cultured in SABM Small Airway Epithelial Cell Growth Basal Medium (Cat# CC-3119). Cells were seeded into 6 well plates for the treatment of 2 ng/ml TGF-β with or without 20 μM GSK4112 (Cat#: 3663; TOCRIS) and SR8278 (Cat#: S9576 Sigma) for 2 days. Human Fetal Lung fibroblast (HFL-1, Cat#: CCL-153) and human bronchial epithelial (BEAS-2B, Cat#: CRL-9609) cells were purchased from the American Type Culture Collection (ATCC) and stored in liquid nitrogen. The cells were thawed and cultured in DMEM/F12K medium (Cat#:113-20033; Thermo Fisher Scientific) with 1% Penicillin-Streptomycin-Glutamine (Cat#: 103-78016; Thermo Fisher Scientific), and 10% FBS (Cat#: 10082147; Thermo Fisher Scientific) for HFL-1 and 1% Penicillin-Streptomycin-Glutamine, 5% FBS for BEAS-2B. Cells were maintained under 5% CO_2_ and 95% humidity. Before treatment, HFL-1 cells were starved in serum-free DMEM/F12K medium for 12 h, and BEAS-2B cells were serum-deprived in DMEM/F12K medium with 1% FBS. Then, the cells were treated with 2 ng/ml TGF-β with or without 20 μM GSK4112 (Cat#: 3663; TOCRIS) and SR8278 (Cat#: S9576 Sigma) for 2 days. After treatment, the cells were either lysed for protein/RNA quantification or fixed with 4% paraformaldehyde for immunofluorescence staining.

### RNA isolation and qRT-PCR

Frozen lungs or cells were homogenized and lysed by QIAzol reagent (Cat#:79306, Qiagen), and mixed with chloroform for 10 s. The mixtures were centrifuged at 12,000 × *g* for 30 min, at 4 °C. Then, the aqueous phase was transferred into a new tube. Equal volumes of isopropanol were added to the samples and mixed universally, then incubated at −20 °C for 2 h. The mixtures were centrifuged at 15,000 × *g* for 15 min at 4 °C, and the supernatants were removed. A total 1 ml 75% EtOH was added to wash the RNA pellet and then spun down at 15,000 × *g*, for 30 min at 4 °C. The EtOH was removed and the RNA precipitates were resuspended in 50 μl of RNase-free water. The concentrations and qualities of all the samples were quantified by Nano-drop spectrophotometer (ND-1000, NanoDrop Technologies). Equal amounts of RNA samples were used for reverse transcription via RT2 First Strand Kit (Cat# 330401, Qiagen) and real-time PCR quantification based on SYBR green expression master-mix (Cat# 330509, Qiagen). The primers used in this study were purchased from BioRad: COL1A1 (Mouse, qMmuCED0044222), FN1 (Mouse, qMmuCEP0054113), TJP1 (Mouse, qMmuCID0005277), TGFB1 (Mouse, qMmuCED0044726), NR1D1 (Mouse, qMmuCID0014284), ARNTL (Mouse, qMmuCED0049609), CLOCK (Mouse, qMmuCED0046959), GAPDH (Mouse, qMmuCEP0039581), COL1A1 (Human, qHsaCEP0050510), ACTA2 (Human, qHsaCIP0028813), FN1 (Human, qHsaCEP0050873), LOX (Human, qHsaCED0043469), LOXL1 (Human, qHsaCED0044245), LOXL2 (Human, qHsaCED0044522), and GAPDH (Human, qHsaCEP0041396). A qRT-PCR thermal cycle is 10 min at 95 °C, 40 cycles of 95 °C, 15 s, and 60 °C, 1 min, the fluorescence intensity was checked at the end of 60 °C incubation. A melting curve was performed for a quality check of cDNA amplification. The BioRad CFX96 qPCR machine was used, and the change fold was calculated based on 2^−ΔΔCt^ methods with GAPDH as the endogenous control.

### NanoString measurement

RNA samples isolated from lungs were used for NanoString measurement with a total of 100 ng RNA for each group. Our customized codeset (circadian genes and fibrotic markers) was used for bleomycin treatment groups, and nCounter Fibrosis Panel was used in Bleomycin + SR9009-treated group as well as IAV infected groups. All the RNA samples were mixed with the master mix and incubated at 65 °C for 16 h for RNA hybridization. All the samples were loaded into a NanoString running cartridge, and profiling reading was performed by nCounter SPRINT Profiler (NanoString Technologies, Inc.). All the gene expressions were normalized by nSolver 4.0 software, and normalized counts were used for data representation. The RLF files generated by the profiler were uploaded to ROSALIND (https://www.rosalind.bio/) for advanced analysis to generate volcano plots and pathway direct enrichment scoring. The significantly dysregulated genes were filtered and uploaded to an online tool (http://bioinformatics.psb.ugent.be/webtools/Venn/) to generate the Venn diagram and overlapped dysregulated gene list.

### Protein isolation and Western blot

Snap frozen lung lobes or cells were lysed in RIPA buffer with a protease inhibitor cocktail, and the protein concentrations were measured by Pierce BCA Assay Kit (Cat#: 23227, Thermo Fisher Scientific). A total 20 µg protein for each sample was used for analysis. The protein samples were separated by 10% sodium dodecyl sulfate–polyacrylamide gel electrophoresis (SDS-PAGE), and then transferred to a nitrocellulose membrane (Cat# 1620112, BioRad). The membranes were then blocked with EveryBlot Blocking Buffer (Cat#: 12010020, BioRad) for 20 min, and incubated with primary antibody diluted in blocking buffer overnight at 4 °C. Primary antibodies used here included anti-REV-ERBα (1:1000, 13418, Cell Signaling), anti-COL4A1 (1:1000, ab227616, Abcam), anti-LOXL2 (1:1000, ab197779, Abcam), anti-E-Cadherin (1:1000, 3195, Cell Signaling), anti-Fibronectin (1:1000, ab, Abcam), anti-vimentin (1:1000, ab92547, Abcam); anti-COL1A2 (1:1000, NBP2-92790, Novus Biologicals), anti-COL1A1 (1:1000, NBP1-30054, Novus Biologicals), anti-activated LOX (1:1000, NB100-2527, Novus Biologicals) for Fig. 7 only, and anti-LOX (1:1000, ab174316, abcam). Then, the primary antibody was removed, and the membranes were washed with Tris-buffered saline containing 0.1% Tween 20 (TBS-T) 3 times, 10 min each. Then, membranes were incubated with secondary antibody (goat-anti-rabbit, 1:5000, #1706515, BioRad) for 1 h at room temperature. The membranes were then washed with TBS-T 4 times, 15 min each. The membranes were developed with Pierce ECL Western Blotting Substrate (Cat#: 32106, Thermo Scientific), and the signals were detected by Bio-Rad ChemiDoc MP imaging system Densitometry was calculated using ImageLab software (BioRad), and fold changes were calculated based on PBS groups, with normalization to β-actin (1:2500, ab20272, Abcam) for mice and GAPDH (1:1000, ab9482, Abcam) for human samples.

### H&E staining

Lung sections (5 µm) were prepared through the microtome, then deparaffinized and rehydrated with xylene, and 100, 95, and 70% EtOH. Then, the sections were stained with hematoxylin for 1 min, rinsed with water for 5 min, and blued with 0.1% ammonia-water for 10 s. The slides were washed with running water for 10 min. Then, the slides were incubated with 95% EtOH for 1 min, then stained with Eosin for 1 min, and quickly washed with 95% EtOH. Then, the slides were sequentially dehydrated with 95%, 100% EtOH, and xylene. Then, all the slides were mounted with Permount, ×4 and ×20 pictures were taken with a light microscope (Nikon ECLIPSE Ci), and the total injured area was measured via ImageJ.

### Immunohistological chemistry (IHC) staining

Lung sections (5 µm) were deparaffinized and rehydrated via xylene, 100, 95, and 70% EtOH, and washed with water for 5 min. Slides were incubated with antigen retrieval solution (Cat#: S1699, Dako, Denmark) at 95 °C for 30 min. Then, the slides were cooled to room temperature and washed with TBS + 0.25% triton-100 (wash buffer) 2 times, 5 min each. Sections were then blocked with 10% normal goat serum and incubated with anti-COL1A1 (1:100, NBP1-30054, Novus Biologicals), anti-Lox (1:100, NB100-2527, Novus Biologicals), anti-Col4A1 (1:200, ab227616, Abcam), and anti-Rev-erbα (1:100, NBP1-84931, Novus Biologicals) at 4 °C overnight. Slides were washed with wash buffer 10 min, 2 times, then incubated with 0.3% hydrogen peroxide for 15 min. Slides were washed with TBS 10 min, 2 times, and washed with wash buffer 5 min, 3 times. Slides were incubated with secondary antibody (1:1000, ab7090, Abcam) at room temperature for 1 h. Then, washed with wash buffer 10 min, 2 times, and developed with DAB Quanto Chromogen and Substrate (Cat#: TA-125-QHDX, Thermo Fisher Scientific) for 10 min. Excess DAB substrate was washed away with water, and counter stained with hematoxylin. Then, the sections were dehydrated and mounted for light microscopy (×20 and ×40 with Nikon ECLIPSE Ci and ×4 with BioTek Cytation 5). All the antibodies were prepared in 10% normal goat serum. ImageJ was used to calculate the positively stained area percentage via color deconvolution.

### Immunofluorescence (IF) staining

Cells were seeded in chamber slides, treated with TGFβ and Rev-erbα agonist/antagonist for 2 days, and then fixed with 4% paraformaldehyde for 15 min. The slides were washed with TBS for 10 min, 2 times, stored at 4 °C, and then blocked with 10% normal goat serum. Cells were incubated anti-COL1A1 (1:100, NBP1-30054, Novus Biologicals) and anti-αSMA (1:200, A2547-2ML, Sigma Life) Sciences at 4 °C overnight, and washed with TBS 3 times, 10 min each. The chamber slides were then incubated with goat anti-rabbit IgG (H + L) secondary antibody Alexa Fluor 488 (1:1000, Catalog # A-11008, Thermo Fisher) and goat anti-Mouse IgG (H + L) Cross-Adsorbed Secondary Antibody-Alexa Fluor 488 (1:1000, Catalog # A-11001, Thermo Fisher) for 1 h at room temperature. Then, cells were washed with TBS 3 times, 15 min each, and the slides were mounted by Diamond Antifade Mountant with DAPI (Cat#: S36964, Fisher Scientific). Slides were imaged by fluorescence microscopy, and ImageJ was used to quantify the fluorescence intensity with the following equation: integrated Density (IntDen) − (Area of cells * Mean fluorescence of background). The intensity was normalized to cell number, and cell number was counted based on DAPI staining via cell counter in ImageJ.

### Statistical analysis

The significant difference was calculated by one-way ANOVA or Student’s *t* test via GraphPad Prism software (V.9.0), and *p* < 0.05 was considered a significant difference. All the data were presented as mean ± SEM.

### Reporting summary

Further information on research design is available in the Nature Portfolio Reporting Summary linked to this article.

## Supplementary information

Supplementary Information

Description of Additional Supplementary Files

Supplementary Data 1

Reporting Summary

## Data Availability

All data support the results of this manuscript are available in the article or Supplementary Information, and raw data will be available upon request. Source data are provided with this paper.

## Source data

Source Data

## References

[1] Huapaya JA, Wilfong EM, Harden CT, Brower RG, Danoff SK (2018). Risk factors for mortality and mortality rates in interstitial lung disease patients in the intensive care unit. Eur. Respir. Rev..

[2] Pain M (2014). Tissue remodelling in chronic bronchial diseases: from the epithelial to mesenchymal phenotype. Eur. Respir. Rev..

[3] Huang WJ, Tang XX (2021). Virus infection induced pulmonary fibrosis. J. Transl. Med..

[4] Antoniou KM (2014). Interstitial lung disease. Eur. Respir. Rev..

[5] Maher TM (2018). Pirfenidone in patients with unclassifiable progressive fibrosing interstitial lung disease: design of a double-blind, randomised, placebo-controlled phase II trial. BMJ Open Respir. Res..

[6] Moua T, Ryu JH (2019). Obstacles to early treatment of idiopathic pulmonary fibrosis: current perspectives. Ther. Clin. Risk Manag..

[7] Durheim MT (2021). Outcomes of patients with advanced idiopathic pulmonary fibrosis treated with nintedanib or pirfenidone in a real-world multicentre cohort. Respirology.

[8] Cunningham PS (2020). The circadian clock protein REVERBα inhibits pulmonary fibrosis development. Proc. Natl Acad. Sci. USA.

[9] Li X, Zhu L, Wang B, Yuan M, Zhu R (2017). Drugs and targets in fibrosis. Front. Pharm..

[10] Liu AC (2008). Redundant function of REV-ERBalpha and beta and non-essential role for Bmal1 cycling in transcriptional regulation of intracellular circadian rhythms. PLoS Genet..

[11] Cho H (2012). Regulation of circadian behaviour and metabolism by REV-ERB-α and REV-ERB-β. Nature.

[12] Solt LA (2012). Regulation of circadian behaviour and metabolism by synthetic REV-ERB agonists. Nature.

[13] Gibbs JE (2012). The nuclear receptor REV-ERBα mediates circadian regulation of innate immunity through selective regulation of inflammatory cytokines. Proc. Natl Acad. Sci. USA.

[14] Albrecht U (2012). Timing to perfection: the biology of central and peripheral circadian clocks. Neuron.

[15] Mohawk JA, Green CB, Takahashi JS (2012). Central and peripheral circadian clocks in mammals. Annu. Rev. Neurosci..

[16] Chang YC (2014). Arecoline-induced myofibroblast transdifferentiation from human buccal mucosal fibroblasts is mediated by ZEB1. J. Cell Mol. Med..

[17] Chomez P (2000). Increased cell death and delayed development in the cerebellum of mice lacking the rev-erbA(alpha) orphan receptor. Development.

[18] Sundar IK, Rashid K, Sellix MT, Rahman I (2017). The nuclear receptor and clock gene REV-ERBα regulates cigarette smoke-induced lung inflammation. Biochem. Biophys. Res. Commun..

[19] Pariollaud M (2018). Circadian clock component REV-ERBα controls homeostatic regulation of pulmonary inflammation. J. Clin. Invest..

[20] Haspel JA (2014). Circadian rhythm reprogramming during lung inflammation. Nat. Commun..

[21] Hwang JW, Sundar IK, Yao H, Sellix MT, Rahman I (2014). Circadian clock function is disrupted by environmental tobacco/cigarette smoke, leading to lung inflammation and injury via a SIRT1-BMAL1 pathway. FASEB J..

[22] Sengupta S (2019). Circadian control of lung inflammation in influenza infection. Nat. Commun..

[23] Giri A, Wang Q, Rahman I, Sundar IK (2022). Circadian molecular clock disruption in chronic pulmonary diseases. Trends Mol. Med..

[24] Gibbs J (2014). An epithelial circadian clock controls pulmonary inflammation and glucocorticoid action. Nat. Med..

[25] Gibbs JE (2012). The nuclear receptor REV-ERBα mediates circadian regulation of innate immunity through selective regulation of inflammatory cytokines. Proc. Natl Acad. Sci. USA.

[26] Sundar IK (2015). Influenza A virus-dependent remodeling of pulmonary clock function in a mouse model of COPD. Sci. Rep..

[27] Wang Q, Sundar IK, Lucas JH, Muthumalage T, Rahman I (2021). Molecular clock REV-ERBα regulates cigarette smoke-induced pulmonary inflammation and epithelial-mesenchymal transition. JCI Insight.

[28] Smith-Mungo LI, Kagan HM (1998). Lysyl oxidase: properties, regulation and multiple functions in biology. Matrix Biol..

[29] Izbicki G, Segel MJ, Christensen TG, Conner MW, Breuer R (2002). Time course of bleomycin-induced lung fibrosis. Int. J. Exp. Pathol..

[30] Wang C, Xuan X, Yao W, Huang G, Jin J (2015). Anti-profibrotic effects of artesunate on bleomycin-induced pulmonary fibrosis in Sprague Dawley rats. Mol. Med. Rep..

[31] Kitzerow O, Zucker IH, Lisco SJ, Wang HJ (2022). Timeline of multi-organ plasma extravasation after bleomycin-induced acute lung injury. Front. Physiol..

[32] Moeller A, Ask K, Warburton D, Gauldie J, Kolb M (2008). The bleomycin animal model: a useful tool to investigate treatment options for idiopathic pulmonary fibrosis?. Int. J. Biochem. Cell Biol..

[33] Cosgrove GP, Bianchi P, Danese S, Lederer DJ (2018). Barriers to timely diagnosis of interstitial lung disease in the real world: the INTENSITY survey. BMC Pulm. Med..

[34] Kalchiem-Dekel O, Galvin JR, Burke AP, Atamas SP, Todd NW (2018). Interstitial lung disease and pulmonary fibrosis: a practical approach for general medicine physicians with focus on the medical history. J. Clin. Med..

[35] Giri A, Rahman I, Sundar IK (2022). Circadian clock-based therapeutics in chronic pulmonary diseases. Trends Pharm. Sci..

[36] Dong C, Gongora R, Sosulski ML, Luo F, Sanchez CG (2016). Regulation of transforming growth factor-beta1 (TGF-β1)-induced pro-fibrotic activities by circadian clock gene BMAL1. Respir. Res..

[37] Pekovic-Vaughan V (2014). The circadian clock regulates rhythmic activation of the NRF2/glutathione-mediated antioxidant defense pathway to modulate pulmonary fibrosis. Genes Dev..

[38] Sundar IK, Yao H, Sellix MT, Rahman I (2015). Circadian molecular clock in lung pathophysiology. Am. J. Physiol. Lung Cell Mol. Physiol..

[39] Lagishetty V (2014). Dysregulation of CLOCK gene expression in hyperoxia-induced lung injury. Am. J. Physiol. Cell Physiol..

[40] Shi Y (2012). Retinoic acid-related orphan receptor-α is induced in the setting of DNA damage and promotes pulmonary emphysema. Am. J. Respir. Crit. Care Med..

[41] Xiong G, Xu R (2022). Retinoid orphan nuclear receptor alpha (RORα) suppresses the epithelial-mesenchymal transition (EMT) by directly repressing Snail transcription. J. Biol. Chem..

[42] Chong NW, Codd V, Chan D, Samani NJ (2006). Circadian clock genes cause activation of the human PAI-1 gene promoter with 4G/5G allelic preference. FEBS Lett..

[43] Cheng T (2014). Lysyl oxidase promotes bleomycin-induced lung fibrosis through modulating inflammation. J. Mol. Cell Biol..

[44] Tjin G (2017). Correction: Lysyl oxidases regulate fibrillar collagen remodelling in idiopathic pulmonary fibrosis (doi: 10.1242/dmm.030114). Dis. Model Mech..

[45] Habermann AC (2020). Single-cell RNA sequencing reveals profibrotic roles of distinct epithelial and mesenchymal lineages in pulmonary fibrosis. Sci. Adv..

[46] Pham TX (2022). Transcriptional analysis of lung fibroblasts identifies PIM1 signaling as a driver of aging-associated persistent fibrosis. JCI Insight.

[47] Dierickx P (2019). SR9009 has REV-ERB-independent effects on cell proliferation and metabolism. Proc. Natl Acad. Sci. USA.

[48] Jouneau S (2020). Analysis of body mass index, weight loss and progression of idiopathic pulmonary fibrosis. Respir. Res..

[49] Kim TH (2022). Impact of body weight change on clinical outcomes in patients with idiopathic pulmonary fibrosis receiving pirfenidone. Sci. Rep..

[50] Elkington PT, Friedland JS (2006). Matrix metalloproteinases in destructive pulmonary pathology. Thorax.

[51] Wang S, Li F, Lin Y, Wu B (2020). Targeting REV-ERBα for therapeutic purposes: promises and challenges. Theranostics.

[52] Welch RD (2017). Rev-Erb co-regulates muscle regeneration via tethered interaction with the NF-Y cistrome. Mol. Metab..

[53] Wollin L (2015). Mode of action of nintedanib in the treatment of idiopathic pulmonary fibrosis. Eur. Respir. J..

[54] Ruwanpura SM, Thomas BJ, Bardin PG (2020). Pirfenidone: molecular mechanisms and potential clinical applications in lung disease. Am. J. Respir. Cell Mol. Biol..

[55] How J (2020). Evaluation of a Pan-lysyl oxidase inhibitor, Pxs-5505, in myelofibrosis: a phase I, randomized, placebo controlled double blind study in healthy adults. Blood.

[56] Li T (2014). Novel role of nuclear receptor Rev-erbα in hepatic stellate cell activation: potential therapeutic target for liver injury. Hepatology.

[57] Amir M (2018). REV-ERBα regulates T(H)17 cell development and autoimmunity. Cell Rep..

[58] Trump RP (2013). Optimized chemical probes for REV-ERBα. J. Med. Chem..

[59] Nogales A, Baker SF, Martínez-Sobrido L (2015). Replication-competent influenza A viruses expressing a red fluorescent protein. Virology.

[60] Rodriguez L, Nogales A, Martínez-Sobrido L (2017). Influenza A virus studies in a mouse model of infection. J. Vis. Exp..

