# Supplementary material for: Circadian clock molecule REV-ERBα regulates lung fibrotic progression through collagen stabilization
Source: Nat Commun. 2023 Mar 9;14:1295. doi: 10.1038/s41467-023-36896-0 (PMC9996598; doi:10.1038/s41467-023-36896-0)
Supplement: Supplementary file 1 — Supplementary Information [file 41467_2023_36896_MOESM1_ESM.pdf]

Supplementary Information

Circadian clock molecule Rev-erbα regulates lung fibrotic progression through collagen stabilization

Authors: [1]Qixin Wang, [2]Isaac Kirubakaran Sundar, [1]Joseph H. Lucas, [3]Jun-Gyu Park,

[3]Aitor Nogales, [3]Luis Martinez-Sobrido, [1]Irfan Rahman*

[1]Department of Environmental Medicine, University of Rochester Medical Center, Rochester, NY, USA

[2]Department of Internal Medicine, Division of Pulmonary, Critical Care and Sleep Medicine, University of Kansas Medical Center, Kansas City, KS, USA

[3]Texas Biomedical Research Institute, Disease Intervention and Prevention Program,

San Antonio, TX 78227, USA.

Address for Correspondence:

* Irfan Rahman, Ph.D.
Department of Environmental Medicine
University of Rochester Medical Center
Box 850, 601 Elmwood Avenue
Rochester 14642, NY, USA
E-mail: irfan_rahman@urmc.rochester.edu

Note: Jun-Gyu Park, PhD is currently affiliated with the Laboratory of Zoonotic Diseases, College of Veterinary Medicine, Chonnam National University, Gwangju, Republic of Korea, and Aitor Nogales, PhD at the Centro de Investigación en Sanidad Animal (CISA-INIA/CSIC), 28130 Madrid, Spain.

Supplementary Figures

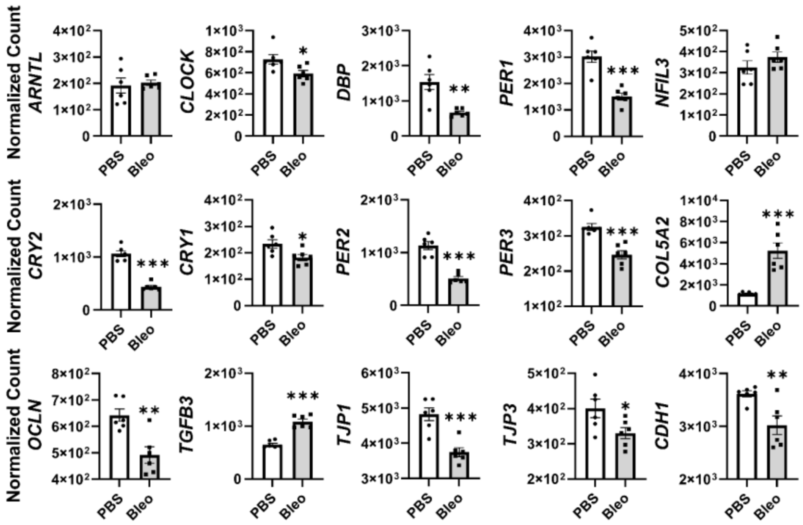

<u>Supplementary Fig. 1.</u> Altered circadian and pro-fibrotic mRNA expressions after bleomycin treatment

Lungs from mice dosed by bleomycin for 14 days. RNA isolated from lung homogenates was used to identify the circadian and profibrotic related gene expressions using our customized nanostring panel through nCounter SPRINT Profiler. The transcripts levels of RNA targets (Normalized Count) were normalized and visualized by nSolver software. Data are shown as mean ± SEM, unpaired 2-side t-test was used. (n=6. * $P < 0.05$, ** $P < 0.01$, *** $P < 0.001$ vs PBS.)

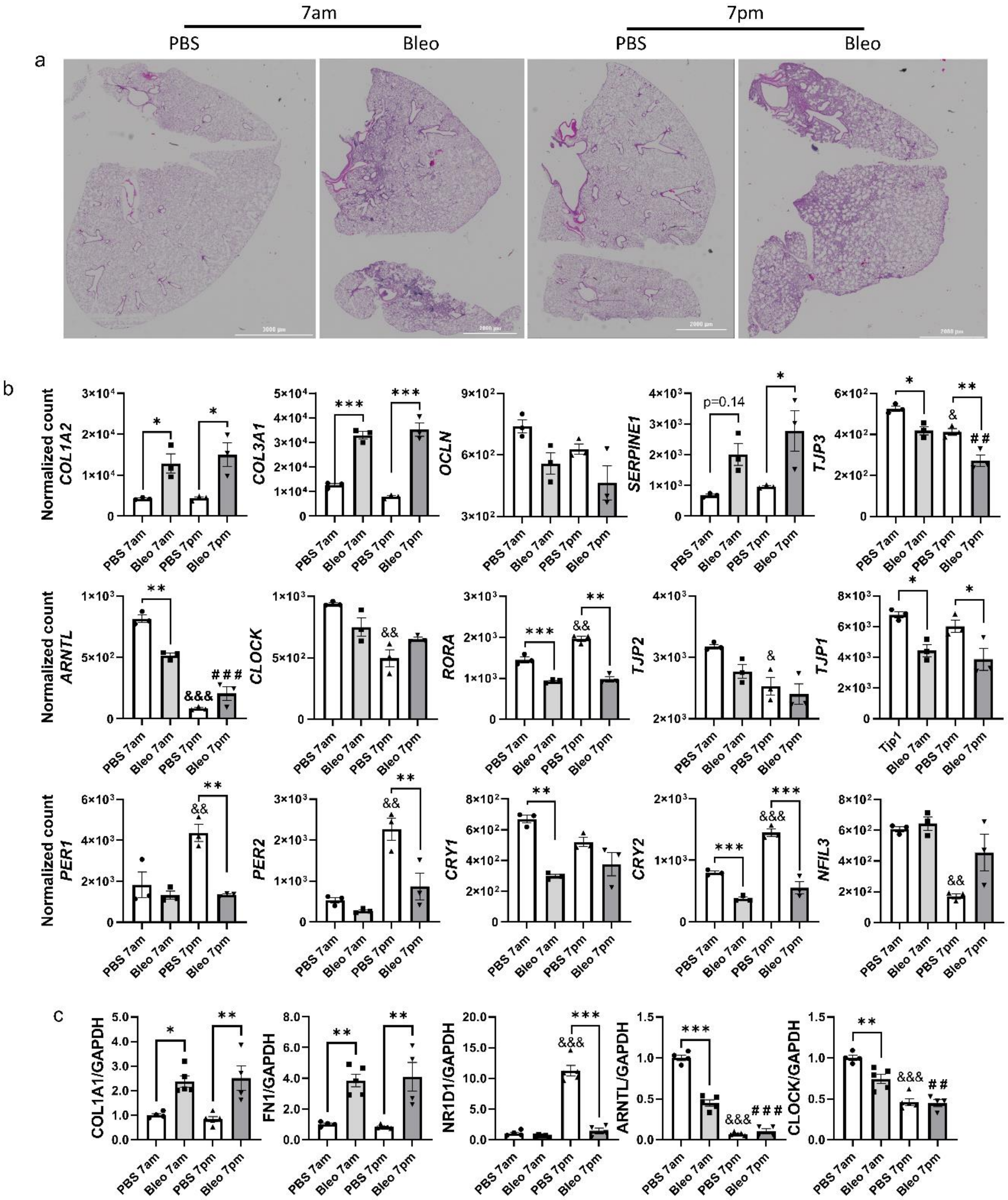

<u>Supplementary Fig. 2.</u> Bleomycin injury at different time points (7 am vs 7 pm) altered circadian and profibrotic mRNA expressions

(a). H&E stained lung sections were visualized and imaged by microscopy. (b). Lungs from mice dosed by bleomycin for 14 days with different time points (7 am and 7 pm) were homogenized for RNA isolation to detect the transcript levels of circadian and profibrotic related gene expressions. Gene measurement was conducted by our customized nanostring panel through nCounter SPRINT Profiler, and transcripts levels were normalized and visualized by nSolver software. (c) The RNA samples were also tested by qRT-PCR, and normalize to GAPDH. Gene expressions were shown as bar graph, and Data were shown as mean ± SEM, one-way ANOVA followed Šídák's multiple comparisons test was used in panel (b-c). Bar size: 3000 µm for PBS 7am group in panel (a), 2000 µm for the rest of figures in panel (a). (n=3. *P < 0.05, **P < 0.01, ***P < 0.001 between groups; # #P<0.01, # # #P<0.01 vs Bleo 7 am group; & & P<0.01, & P<0.001 vs PBS 7 am group).

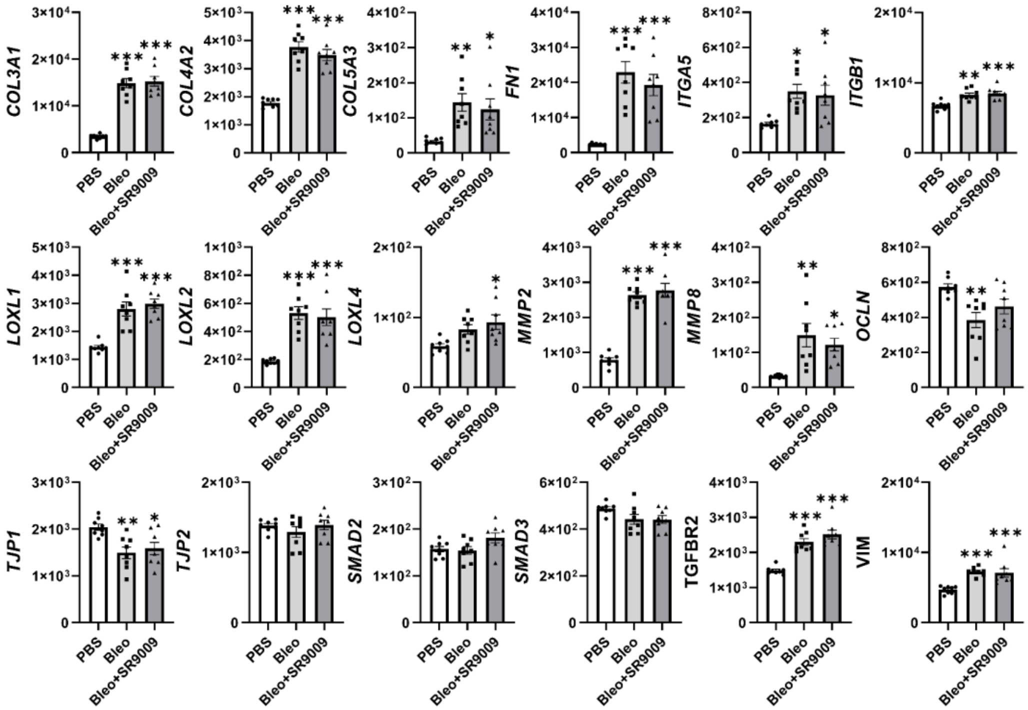

<u>Supplementary Fig. 3.</u> Altered mRNA expressions from bleomycin treated mice with or without SR9009

RNA isolated from lung homogenates was used to identify the gene expressions using the nCounter Fibrosis panel through nCounter SPRINT Profiler. The transcripts levels of RNA targets (Normalized Count) were normalized and visualized by nSolver software. Data are shown as mean ± SEM, one-way ANOVA followed Šídák's multiple comparisons test was used. (n=8, * $P < 0.05$, ** $P < 0.01$, *** $P < 0.001$ vs PBS)

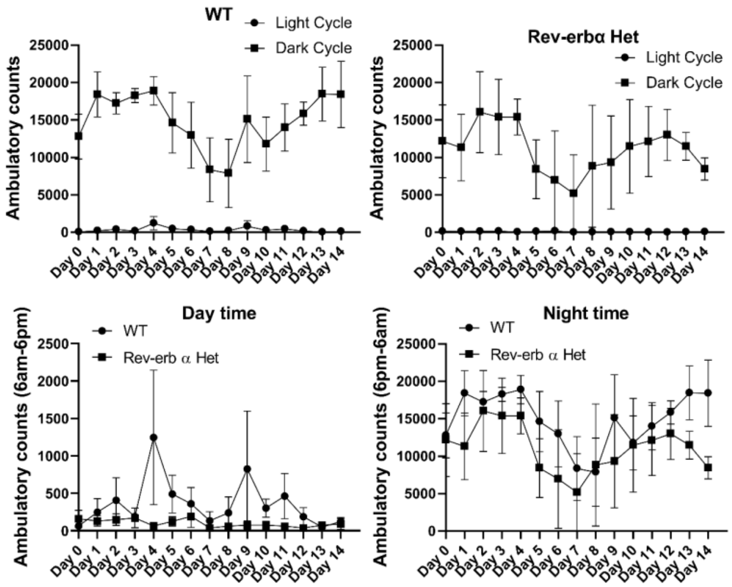

<u>Supplementary Fig. 4.</u> Effect of IAV infection on wheel running activity

After IAV infection (10^3 PFU/mouse), mice were housed in cages with running wheels connected with automatic counter. Mice were entrained in a 12:12 light/Dark cycle during the whole progress. Data are shown as mean ± SEM. (n=2-3 mice per group).

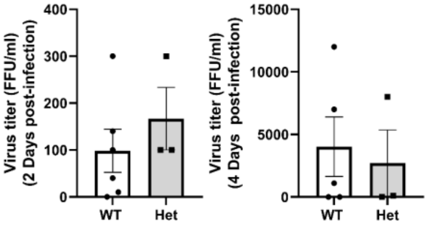

<u>Supplementary Fig. 5.</u> Viral titers at 2 and 4 days post-infection with IAV in mice lungs
WT and Rev-erbα Het mice were with IAV (10^3 PFU/mouse) with IAV. At days 2 and 4 post-infection, lungs were collected and homogenized for viral titer calculation using standard fluorescent assay (fluorescent forming units, FFU/ml). Data are shown as mean ± SEM (n=3-6 mice per group).

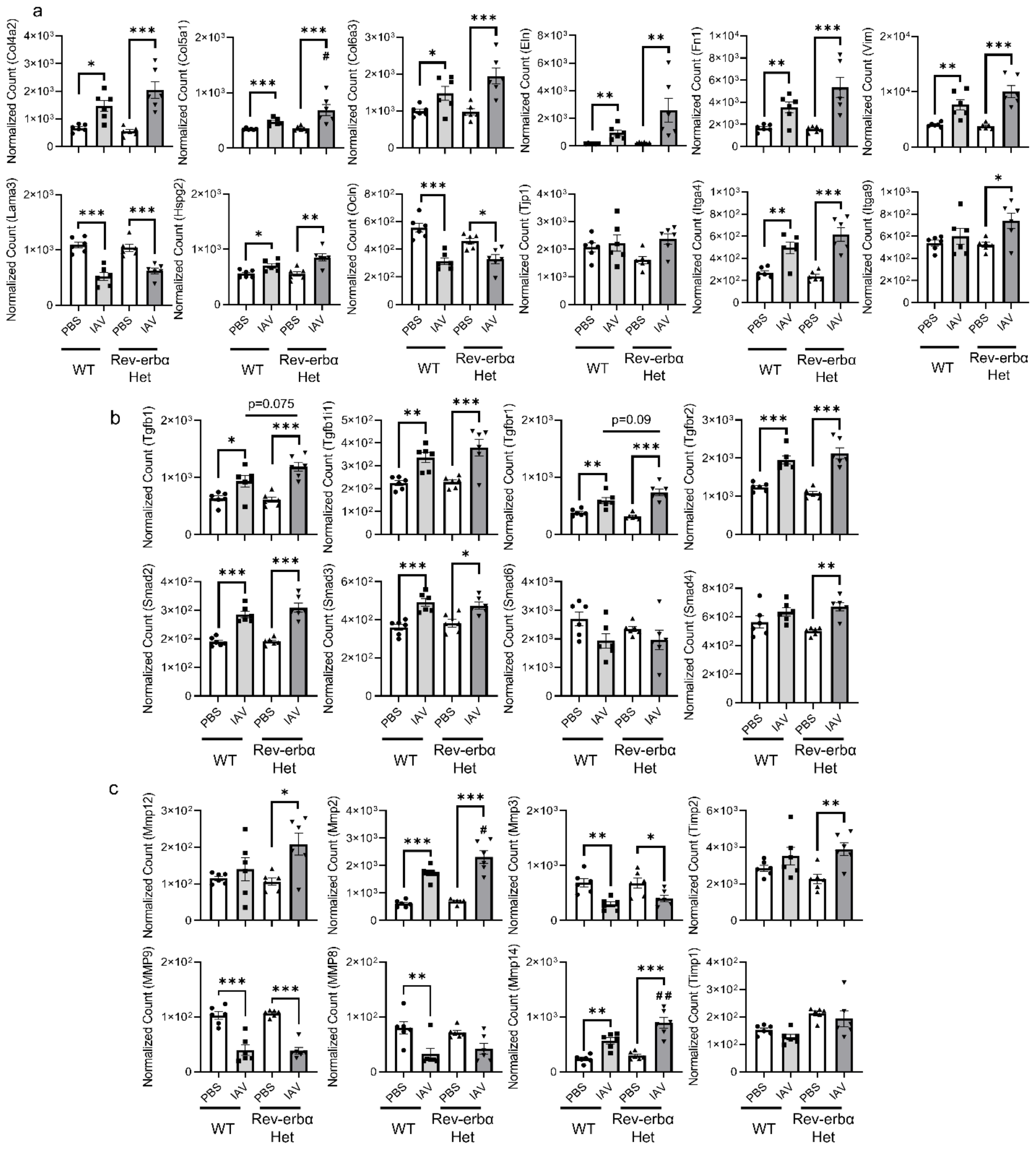

<u>Supplementary Fig. 6</u>. IAV infection induced dysregulation of profibrotic gene expression exacerbated in Rev-erbα Het mice.

WT and Rev-erbα Het mice dosed with IAV (10^3 PFU) for 15 days, and lungs were homogenized for RNA isolation. Gene expressions were measured by nCounter Fibrosis Panel via nCounter SPRINT Profiler. RNA count normalization and analysis were performed via nSolver software. The dysregulated gene expression focused on (A) ECM related genes, (B) TGFβ pathway related genes, and (C) MMPs related genes, which were shown with normalized count in bar graphs. Data are shown as mean ± SEM, one-way ANOVA followed Šídák's multiple comparisons test was used in panel (a), unpaired 2-side t-test was used in panel (a, *COL5A1*, *COL6A3, ELN, FN1, HSPG2* PBS WT vs IAV WT.), panel (b *TGFB1* IAV WT vs IAV Rev-erbα Het). (n = 6; *P < 0.05, **P < 0.01, ***P < 0.001 between groups; $^{\#}$ P < 0.05, $^{\#\#}$ P < 0.01 compared with IAV infected WT group).

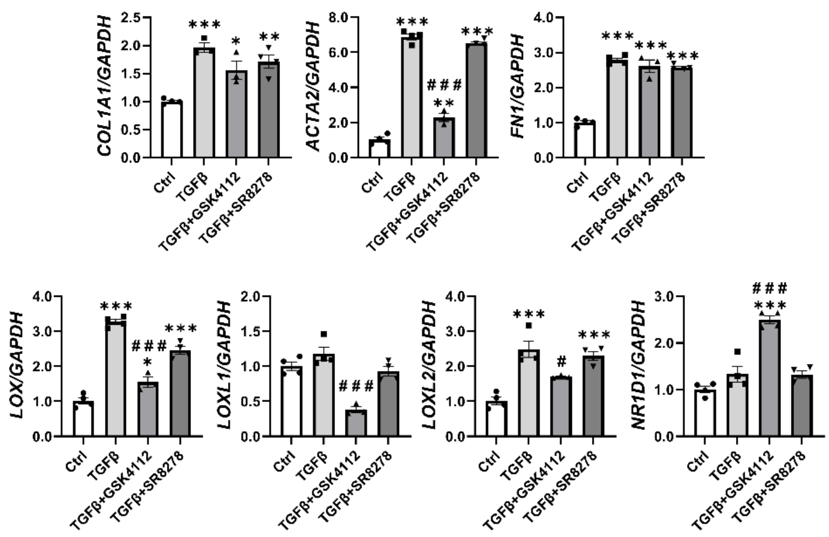

<u>**Supplementary Fig. 7**</u>**. Rev-erbα agonist inhibited TGFβ induced fibroblast differentiation and lysyl oxidase mediated collagen dysregulation while its antagonist showed no effects.**
HFL-1 cells were treated with TGFβ (2 ng/mL) with or without REV-ERBα agonist (GSK4112) and antagonist (SR8278) at 20 µM for 2 days. RNA was isolated for gene expression measurement via qPCR (*COL1A1*, *ACTA2*, *FN1*, *LOX*, *LOXL1*, *LOXL2*, and *NR1D1*). GAPDH was used as an endogenous control for normalization. Data are shown as mean ± SEM, one-way ANOVA followed Šídák's multiple comparisons test was used (n=3-4 per cells, *P < 0.05, **P < 0.01, ***P < 0.001, vs Ctrl group; $^{\#}P < 0.05$, $^{\#\#\#}P < 0.001$, vs TGFβ group).

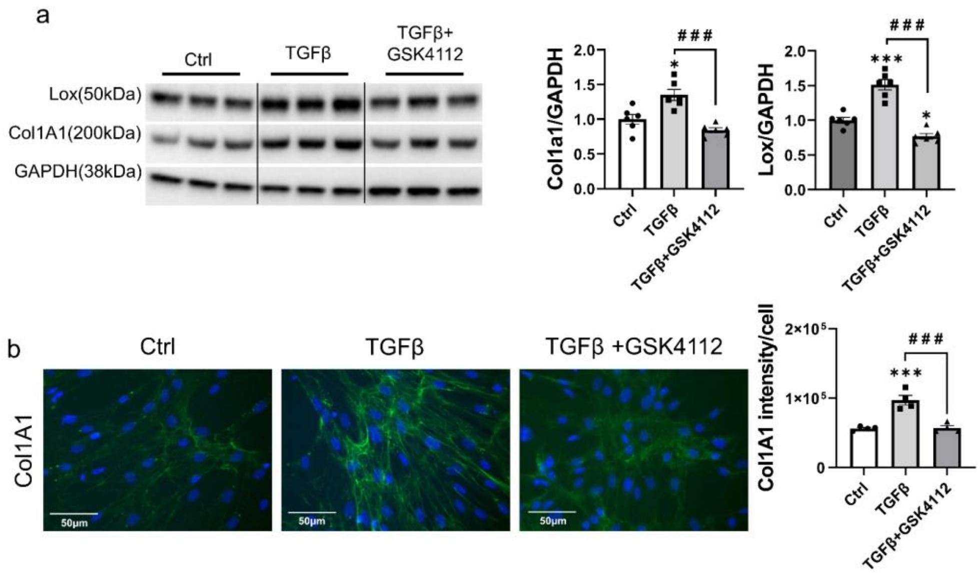

<u>Supplementary Fig. 8</u>. Rev-erbα agonist inhibits TGFβ induced lung fetal fibroblast differentiation and lysyl oxidase-mediated collagen dysregulation.

HFL-1 cells were treated with TGFβ (2 ng/mL) with or without REV-ERBα agonist (GSK4112, 20μM) for 2 days. (a) Protein was isolated for Western blot analysis (Col1A1 and Lox). Represented blots are shown with densitometry analysis. GAPDH was used as an endogenous control for normalization (n=6 cells per group). (b). Immunofluorescence staining showed the distribution and protein abundance of COL1A1. DAPI was used for nuclear staining (20x) (n=4 cells per group). Relative fluorescence intensity was calculated in ImageJ, as fluorescence intensity per cell. Data are shown as mean ± SEM, one-way ANOVA followed Šídák's multiple comparisons test was used for panel (a-b) (*P < 0.05, **P < 0.01, ***P < 0.001, vs Ctrl group; $^{\#}P$ < 0.05, $^{\#\#\#}P$ < 0.001, vs TGFβ group).

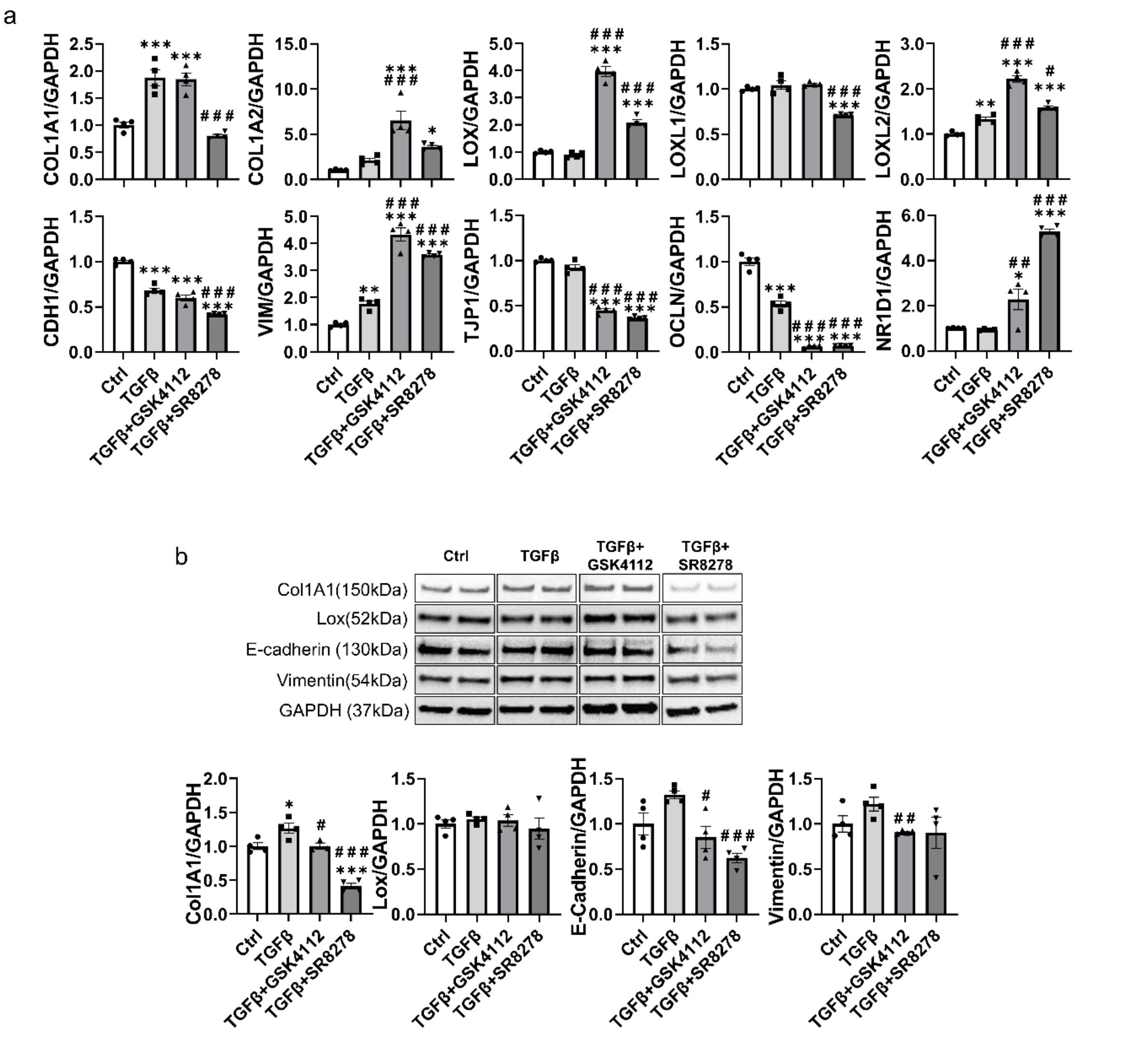

<u>Supplementary Fig. 9</u>. Rev-erbα agonist and antagonist both exacerbated TGFβ induced EMT in SAEC (Small airway epithelial cells)

SAEC were treated with TGFβ (2 ng/mL) with or without REV-ERBα agonist (GSK4112, 20µM) and antagonist (SR8278, 20µM) for 2 days. (a). RNA was isolated for gene expression analysis via qPCR (*COL1A1*, *COL1A2, LOX*, *LOXL1, LOXL2, CDH1, VIM, TJP1, OCLN,* and *NR1D1*) (n=4 cells per group). (b). Protein was isolated for western blotting of Col1A1, Lox, E-cadherin, and Vimentin and analyzed. GAPDH was used as an endogenous control for both gene and protein abundance normalization (n=3-4 cells per group). Data are shown as mean ± SEM, one-way ANOVA followed Šídák's multiple comparisons test was used for panel (a-b), unpaired 2-side t-test was used for panel (b, Vimentin TGFβ vs TGFβ+GSK4112). (**P* < 0.05, ***P* < 0.01, ****P* < 0.001, vs Ctrl group; $^{\#}P$ < 0.05, $^{\#\#}P$ < 0.01, $^{\#\#\#}P$ < 0.001, vs TGFβ group).

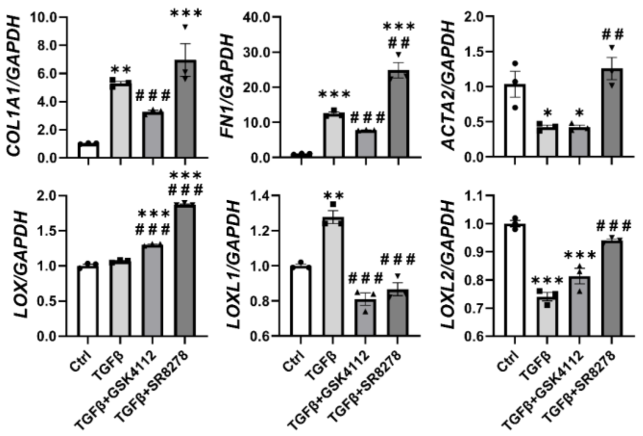

Supplementary Fig. 10. Rev-erbα agonist inhibited TGFβ induced EMT in lung epithelium cells while antagonist exacerbated the expression of *LOX* and *FN1*

BEAS-2B cells were treated with TGFβ (2 ng/mL) with or without REV-ERBα agonist (GSK4112, 20 µM) and antagonist (SR8278, 20 µM) for 2 days. RNA was isolated for gene expression measurement via qPCR (*COL1A1*, *ACTA2*, *FN1*, *LOX*, *LOXL1,* and *LOXL2*). GAPDH was used as an endogenous control for normalization (n=3 cells per group). Data are shown as mean ± SEM, one-way ANOVA followed Šídák's multiple comparisons test was used for all panels, unpaired 2-side t-test was performed for panel (*COL1A1*, TGFβ vs TGFβ+GSK4112). (**P* < 0.05, ****P* < 0.001, vs Ctrl group; $^{\#}P < 0.05$, $^{\#\#\#}P < 0.001$, vs TGFβ group).

Supplementary Table 1. Patient information for healthy control and IPF patient

	Age	Gender	Pathology Verification	Appearance	Normal%	Lesion%	Tumor%	Sample ID	Pathology Verification Notes from H&E review
Ctrl-1	69	Male	Within normal limits	Normal	100	0	0	PA00003E25	90% Alveoli, 5% Bronchioles, 5% Fibrovascular septa
Ctrl-2	54	Male	Within normal limits	Normal	100	0	0	PA000061F4	100% alveoli
Ctrl-3	69	Male	Within normal limits	Normal	100	0	0	PA00003E82	85% alveoli, 5% bronchi, 10% fibrovascular septa
Ctrl-4	72	Female	Within normal limits	Normal	100	0	0	PA15476798	80% alveoli, 0% bronchioles, 20% fibrovascular septae
Ctrl-5	69	Female	Within normal limits	Normal	100	0	0	PA0000160B	95% alveoli, 1% bronchioles, 4% fibrovascular tissue
Ctrl-6	52	Female	Within normal limits	Normal	100	0	0	PA15476DC1	75% alveoli, 5% bronchioles, 20% Fibrovascular septa; 1+ MAC
Ctrl-7	61	Male	Within normal limits	Normal	100	0	0	PA15477B6B	80% Alveoli, 1% Bronchioles, 19% Fibrovascular septa
Ctrl-8	67	Female	Within normal limits	Normal	100	0	0	PA15477FDC	80% Alveoli, 10% Bronchioles, 10% Fibrovascular septa
Ctrl-9	65	Male	Within normal limits	Normal	100	0	0	PA15478781	85% Alveoli, 5% Bronchioles, 10% Fibrovascular septa
Ctrl-10	40	Male	Within normal limits	Normal	100	0	0	PA1547862B	90% Alveoli, 5% Bronchioles, 5% Fibrovascular septa
IPF-1	43	Male	Fibrosis	Lesion	50	50	0	PA0000401B	50% normal component consists of alveoli; sample contains fibrosis
IPF-2	66	Female	Fibrosis	Lesion	0	100	0	PA00000648	Lesion (100%): Chemotherapy effect 100%; Other Features/Comments: No normal architecture in lesional component
IPF-3	62	Male	Fibrosis	Lesion	0	100	0	PA00005059	Lesion (100%): end stage honeycomb lung 100%; Other Features/Comments: No normal architecture in lesional component
IPF-4	54	Male	Fibrosis	Lesion	0	100	0	PA00009ACC	Inflammation: Moderate Macrophages; Other Features/Comments: No normal architecture in lesional component
IPF-5	75	Female	Fibrosis	Lesion	0	100	0	PA15477964	100% No normal architecture
IPF-6	57	Male	Fibrosis	Lesion	60	40	0	PA000087A2	70% Alveoli, 5% Bronchioles, 25% Fibrovascular septa
IPF-7	70	Female	Fibrosis	Lesion	0	100	0	PA0F333964	60% alveoli, 10% bronchioles, 40% fibrovascular septae; contains interstitial fibrosis and chronic inflammation
IPF-8	68	Male	Fibrosis	Lesion	50	50	0	PA00004D63	55% Alveoli, 5% Bronchioles, 20% Fibrovascular septa, 20% Intrapulmonary lymph node
IPF-9	66	Male	Fibrosis	Lesion	58	42	0	PA000019D2	50% Alveoli, 5% Bronchioles, 3% Fibrovascular tissue, 42% Diffuse interstitial fibrosis
IPF-10	51	Male	Fibrosis	Lesion	20	80	0	PA0F3348D3	Inflammation: Moderate Mixed inflammatory infiltrate; 65% Alveoli, 15% Bronchioles, 20% Fibrovascular septa
