# Supplementary material for: Circadian clock molecule REV-ERBα regulates lung fibrotic progression through collagen stabilization
Source: Nat Commun. 2023 Mar 9;14:1295. doi: 10.1038/s41467-023-36896-0 (PMC9996598; doi:10.1038/s41467-023-36896-0)
Supplement: Supplementary file 2 — Description of Additional Supplementary Files [file 41467_2023_36896_MOESM2_ESM.pdf]

Description of Additional Supplementary Files

Supplementary Data 1: Dysregulated gene lists among different comparison.
