# Supplementary material for: Circadian clock molecule REV-ERBα regulates lung fibrotic progression through collagen stabilization
Source: Nat Commun. 2023 Mar 9;14:1295. doi: 10.1038/s41467-023-36896-0 (PMC9996598; doi:10.1038/s41467-023-36896-0)
Supplement: Supplementary file 4 — Reporting Summary [file 41467_2023_36896_MOESM4_ESM.pdf]

nature portfolio

Corresponding author(s): Irfan Rahman

Last updated by author(s): Feb 15, 2023

Reporting Summary

Nature Portfolio wishes to improve the reproducibility of the work that we publish. This form provides structure for consistency and transparency in reporting. For further information on Nature Portfolio policies, see our Editorial Policies and the Editorial Policy Checklist.

Statistics

For all statistical analyses, confirm that the following items are present in the figure legend, table legend, main text, or Methods section.

n/a	Confirmed	
[ ]	[x]	The exact sample size (*n*) for each experimental group/condition, given as a discrete number and unit of measurement
[ ]	[x]	A statement on whether measurements were taken from distinct samples or whether the same sample was measured repeatedly
[ ]	[x]	The statistical test(s) used AND whether they are one- or two-sided<br>*Only common tests should be described solely by name; describe more complex techniques in the Methods section.*
[x]	[ ]	A description of all covariates tested
[ ]	[x]	A description of any assumptions or corrections, such as tests of normality and adjustment for multiple comparisons
[ ]	[x]	A full description of the statistical parameters including central tendency (e.g. means) or other basic estimates (e.g. regression coefficient) AND variation (e.g. standard deviation) or associated estimates of uncertainty (e.g. confidence intervals)
[ ]	[x]	For null hypothesis testing, the test statistic (e.g. *F*, *t*, *r*) with confidence intervals, effect sizes, degrees of freedom and *P* value noted<br>*Give P values as exact values whenever suitable.*
[x]	[ ]	For Bayesian analysis, information on the choice of priors and Markov chain Monte Carlo settings
[x]	[ ]	For hierarchical and complex designs, identification of the appropriate level for tests and full reporting of outcomes
[x]	[ ]	Estimates of effect sizes (e.g. Cohen's *d*, Pearson's *r*), indicating how they were calculated

Our web collection on statistics for biologists contains articles on many of the points above.

Software and code

Policy information about availability of computer code

Data collection	Nikon Eclipse and Spot Software 5.0 were used to take microscope images; Gen5 version: 3.01.17 installed in Cytation 5 imaging system was used to take whole scanning of sections.
Data analysis	1. Image J (Version: 1.53t) was used to perform the data analysis especially on positive staining area analysis via the function of color deconvolution.<br>2. Nanostring nSolver 4.0 software was used to perform normalization and expression measurement of transcript levels. Heatmap was generated by nSolver software with fixed gene list and condition group orders.<br>3. The gene pathway analysis was performed by Rosalind (https://www.rosalind.bio/), the volcano plots were generated by Rosalind as well.<br>4. The significantly dysregulated gene list and the venn diagrams were generated and analyzed by http://bioinformatics.psb.ugent.be/webtools/Venn.

For manuscripts utilizing custom algorithms or software that are central to the research but not yet described in published literature, software must be made available to editors and reviewers. We strongly encourage code deposition in a community repository (e.g. GitHub). See the Nature Portfolio guidelines for submitting code & software for further information.

Data

Policy information about availability of data

All manuscripts must include a data availability statement. This statement should provide the following information, where applicable:

- Accession codes, unique identifiers, or web links for publicly available datasets
- A description of any restrictions on data availability
- For clinical datasets or third party data, please ensure that the statement adheres to our policy

The un-cropped full blot for western blot analysis were presented individually in support information, and all the data used for generating figures were provided in the source data excel file.

Human research participants

Policy information about studies involving human research participants and Sex and Gender in Research.

Reporting on sex and gender	We do not perform human subject in this study, the human samples we have included in this study were purchased from Origene Inc, and the patient information provided in the Table S1 is based on the information provided by Origene Inc.
Population characteristics	N/A
Recruitment	N/A
Ethics oversight	N/A

Note that full information on the approval of the study protocol must also be provided in the manuscript.

Field-specific reporting

Please select the one below that is the best fit for your research. If you are not sure, read the appropriate sections before making your selection.

☒ Life sciences ☐ Behavioural & social sciences ☐ Ecological, evolutionary & environmental sciences

For a reference copy of the document with all sections, see nature.com/documents/nr-reporting-summary-flat.pdf

Life sciences study design

All studies must disclose on these points even when the disclosure is negative.

Sample size	At least N=3 mice per group, and N=4 for in vitro experiment, were used to perform the experiments, and we have chosen at least N=5 for human sample analysis.
Data exclusions	No data were excluded from the analysis
Replication	All attempts at replication were successful for the experiments. The experiments were performed independently. All light microscopy images were captured single blinded and individually, at least 10 pictures were used to perform the analysis.
Randomization	Animals were randomly allocated into different groups with similar number of male/female if allowed, or just female mice used for Fig 3 and Supplementary Fig 2. Human samples were randomly picked up from our inventory, which we purchased from Origene Inc.
Blinding	IHC and ICC data were captured and analyzed single-blindly, while the control group is hard to be blinded, since the condition was relatively evident in each group. The investigators were blinded to groups during data collection among specific groups, such as the sex difference and genotype difference, while analysis was not blinded.

Reporting for specific materials, systems and methods

We require information from authors about some types of materials, experimental systems and methods used in many studies. Here, indicate whether each material, system or method listed is relevant to your study. If you are not sure if a list item applies to your research, read the appropriate section before selecting a response.

Materials & experimental systems

n/a	Involved in the study
☐	☒ Antibodies
☐	☒ Eukaryotic cell lines
☒	☐ Palaeontology and archaeology
☐	☒ Animals and other organisms
☒	☐ Clinical data
☒	☐ Dual use research of concern

Methods

n/a	Involved in the study
☒	☐ ChIP-seq
☒	☐ Flow cytometry
☒	☐ MRI-based neuroimaging

Antibodies

Antibodies used	For western blot<br>anti-vimentin (1:1000, ab92547, Abcam);<br>anti-COL1A2 (1:1000, NBP2-92790, Novus Biologicals),<br>anti-COL1A1 (1:1000, NBP1-30054, Novus Biologicals),<br>activated Lox (1:1000, NB100-2527, Novus Biologicals)<br>Lox (1:1000, ab174316, abcam)<br>β-actin (1:2500, ab20272, Abcam)<br>GAPDH (1:1000, ab9482, Abcam)<br>secondary antibody (goat-anti-rabbit, 1:5000, #1706515, BioRad)<br><br>For IHC measurement<br>anti-COL1A1 (1:100, NBP1-30054, Novus Biologicals),<br>anti-Lox (1:100, NB100-2527, Novus Biologicals),<br>anti-Rev-erbα (1:100, NBP1-84931, Novus Biologicals)<br>Anti-Col4A1 (1:200, ab227616, Abcam)<br>secondary antibody (1:1000, ab7090, Abcam)<br><br>For ICC measurement<br>anti-COL1A1 (1:100, NBP1-30054, Novus Biologicals)<br>anti-Actin, α-Smooth Muscle (1:200, A2547-2ML, Sigma Life)<br>goat anti-rabbit IgG (H+L) secondary antibody Alexa Fluor™ 488 (1:1000, Catalog # A-11008, ThermoFisher)<br>Goat anti-Mouse IgG (H+L) Cross-Adsorbed Secondary Antibody, Alexa Fluor™ 488 (1:1000, Catalog # A-11001, ThermoFisher)
Validation	All antibody were validated by the suppliers.<br>Specifically, the identification of mature form of Lox is referred to the previous publication: PMID: 16251195

Eukaryotic cell lines

Policy information about cell lines and Sex and Gender in Research

Cell line source(s)	HFL-1 is purchased from ATCC, Cat#: CCL-153™<br>BEAS-2B is purchased from ATCC, Cat#: CRL-9609<br>Primary Lung fibroblast (HLF) is purchased from Lonza Inc, Catalog #: CC-2512<br>Small airway epithelial cell (SAEC) is purchased from Lonza Inc, Catalog #: CC-2547
Authentication	Cell lines and primary cells were directly purchased from ATCC or Lonza, and cultured in proper growth medium per company suggestion The cells were stocked in early passage and performed experiments individually.
Mycoplasma contamination	Cells were not tested for mycoplasma contamination, while no sign of contamination was observed during experiments.
Commonly misidentified lines<br>(See ICLAC register)	No commonly misidentified cell lines were used.

Animals and other research organisms

Policy information about studies involving animals; ARRIVE guidelines recommended for reporting animal research, and Sex and Gender in Research

Laboratory animals	C57BL/6J and Rev-erbα global heterozygous mice (Strain #:018447) were purchased from Jackson Laboratory and bred in vivarium in URMC.
Wild animals	No wild animals were used in this study

Reporting on sex	We used only female C57BL/6J mice for Fig 3 and supplementary Fig 2, we have used similar number (2-3 per group, or 4-6 per group) of male and female mice for Fig 2, 4, 5, 6, and 7, and its respective support figs.
Field-collected samples	No field collection was performed in this study
Ethics oversight	Mouse experiments and its procedures were performed under the approval from Animal Research Committee of the University of Rochester (UCAR, protocol ID: UCAR-2007-070E).

Note that full information on the approval of the study protocol must also be provided in the manuscript.
